# State-of-the-Art Review on Finite Element Analysis Applied to Ultrathin-Strut Drug-Eluting Stents: Examining the Present and Looking Ahead to the Future

**DOI:** 10.3390/bioengineering13070845

**Published:** 2026-07-22

**Authors:** Francesco Nappi, Franck Digne

**Affiliations:** 1 Department of Cardiac Surgery, Centre Cardiologique du Nord, 32-35 Avenue des Moulins Gemeaux, 93200 Saint-Denis, France; 2 Department of Interventional Cardiology, Centre Cardiologique du Nord, 32-35 Avenue des Moulins Gemeaux, 93200 Saint-Denis, France; franck.digne@hotmail.com

**Keywords:** drug-eluting stents, percutaneous coronary intervention, ultrathin struts DESs, computational modeling

## Abstract

The latest drug-eluting stents (DESs) are the gold standard for patient treatment during percutaneous coronary intervention (PCI). The latest advancements in DES innovation have resulted in the development of new stent technologies with reduced thickness in the struts. The new DES design, ultrathin-strut DESs, features struts measuring less than 70 μm in thickness. The evidence for these devices is derived from observational studies, extensive meta-analyses, and randomized trials with long-term outcomes. The investigation is focused on determining the comparative performance of ultrathin-strut DESs and conventional new-generation DESs across various clinical settings and patient lesion profiles. The objective of the seminar is to examine recent advancements in the use of very thin DESs and the potential of computational modeling in coronary arteries during PCI. An analysis of the mechanical performance of ultrathin DESs has been conducted in terms of radial expansion and stresses within the stent-vessel system. Residual stresses generated by the crimping process will also be considered.

## 1. Introduction

Finite element analysis (FEA) is a pivotal approach in acquiring critical data concerning intricate real-world systems. These systems cannot be measured using conventional methods. In the field of medical device design, finite element analysis (FEA) is a crucial tool for calculating stresses and investigating potential sources of failure. This analysis helps identify areas of vulnerability and improves the reliability of the device [1,2,3].

Percutaneous coronary intervention (PCI) is a widely utilized treatment for coronary artery stenoses, which are conditions that restrict blood flow. PCI is a field that is undergoing continuous development, primarily due to the advancement, improvement, and refinement of associated technologies and devices [4]. Currently, the amount of data available on normal coronary artery anatomy, incorporating bifurcations, is very limited, and the accessible information has important restrictions [5,6,7,8,9,10,11]. A number of studies have previously been conducted on coronary anatomy [12,13,14,15,16]. It is important to note that there are a number of discrepancies between the studies. The aforementioned discrepancies encompass, but are not limited to, the inclusion criteria, imaging methodology, angles, definitional framework, and measurement approach [17,18]. To make the best choices for stents, professionals must be familiar with the dimensions of the arteries, the way they look, and how they can change. This knowledge is essential for selecting the most appropriate stent design, thus improving patient outcomes [19]. To enable fluid-dynamic simulations and generate exact 3D coronary-artery prototypes for bench-testing equipment and methods, a precise luminal surface shape is essential [20,21].

Creation of a three-dimensional (3D) computational atlas of coronary artery morphology using computed tomography (CT) represents a significant advancement in the domain of PCI procedures. This development is consistent with the introduction of ultrathin stents, which allow for precise positioning and expansion of the device. Studies on manual projection planes, which minimize vessel shortening, have shown that the bifurcation has a two-dimensional spatial configuration, despite evidence indicating its actual three-dimensional nature. Today, the mechanical and fluid dynamic behavior of the new ultrathin-strut (UtS) drug-eluting stent (DES) can benefit from the use of three-dimensional spatial configurations of the coronary arteries. Given these particular geometric configurations, finite element analysis (FEA) can be utilized to assess the mechanical properties of the UtS-DES. Furthermore, the FEA and computational fluid dynamics analysis will be used to compare the new devices with the most advanced and widely implanted models.

The transition from early-generation to new-generation stents has led to significant advancements in the design of metallic stents, resulting in a wide range of structural innovations [22,23]. Bare-metal stents (BMSs) are typically manufactured using stainless-steel materials. In contrast, the majority of novel-generation DESs utilize a cobalt–chromium alloy (CoCr) or a platinum–chromium alliance (PtCr) metallic framework. The most common DESs used so far have used non-degradable polymer coatings with anti-proliferative medications to deal with the well-documented issue of in-stent restenosis (ISR), a major complication that can result from the use of bare-metal stents [24].

These enhancements have enabled a substantial reduction in the thickness of the struts, from 130–140 µm to 60–80 µm. This modification led to two key benefits: enhanced deliverability and accelerated endothelial coverage following implantation. It is clear that there are differences between the various platforms regarding the anti-proliferative agent and the dosage of the drug. Recent advancements in stent technology have led to the development of stents that utilize a reduced dosage of pharmaceutical agents to promote the process of endothelization. These stents feature a “limus” derivative, which functions as an anti-proliferative agent. Examples of this class of drugs include sirolimus, everolimus, zotarolimus, novolimus, Biolimus, and Umirolimus. The primary purpose of DESs was to decrease the occurrence of neointimal proliferation and restenosis following PCI with BMSs. However, unlike BMSs, early-generation DESs have demonstrated that the evascularity of their design is associated with an increased likelihood of ST [25]. According to the definitions provided by the ARC ST, any death occurring during the follow-up period following a PCI procedure with an unknown cause is classified as a probable (if within 30 days) or possible (beyond 30 days) case of stent thrombosis, employing the same logic outlined above for cardiac death [26].

To address this issue, novel-generation DESs have been developed. These DESs incorporate enhanced biocompatibility of the enduring polymer coating or biodegradable copolymer, in conjunction with evasive pharmaceutical compound safety [27,28]. Recent studies have indicated that new-generation DESs have demonstrated superiority over BMSs in a range of key endpoints, including cardiac death, myocardial infarction (MI), ST, and repeat revascularization [27,29]. Consequently, contemporary DESs are currently recommended for all patients scheduled to undergo PCI, regardless of the projected course of treatment with dual antiplatelet medication [30]. 

Advancements in device engineering in recent years have contributed to a notable reduction in the thickness of struts, with the emergence of a new category called UtS-DESs. The lack of a universal characterization of ultrathin DESs necessitates a delineation of the devices as those with a strut thickness of fewer than seventy micrometers. The use of these devices offers the potential to deliver several benefits, including improved deliverability, reduced vessel injury and disturbance to the flow of side branches. Research indicates that disruption to the flow of side branches may contribute to restenosis following stent implantation.

The review will examine recent advancements in ultrathin DESs and the potential of computational modeling in enhancing coronary artery analysis. The objective of the investigation is to ascertain whether improving the definition of shape variations, encompassing diameters and angles, can ensure more precise stent positioning during PCI, ultimately mitigating potential complications. Finally, the mechanical performance of ultrathin DESs has been analyzed in terms of radial expansion and stresses within the stent-vessel system. A comparison has been made between this product and the previous generation of DESs, which is notable for its absence of polymer. In particular, the results regarding residual stresses generated by the crimping process will be given due consideration.

### Search Strategy

**Figure 1:**
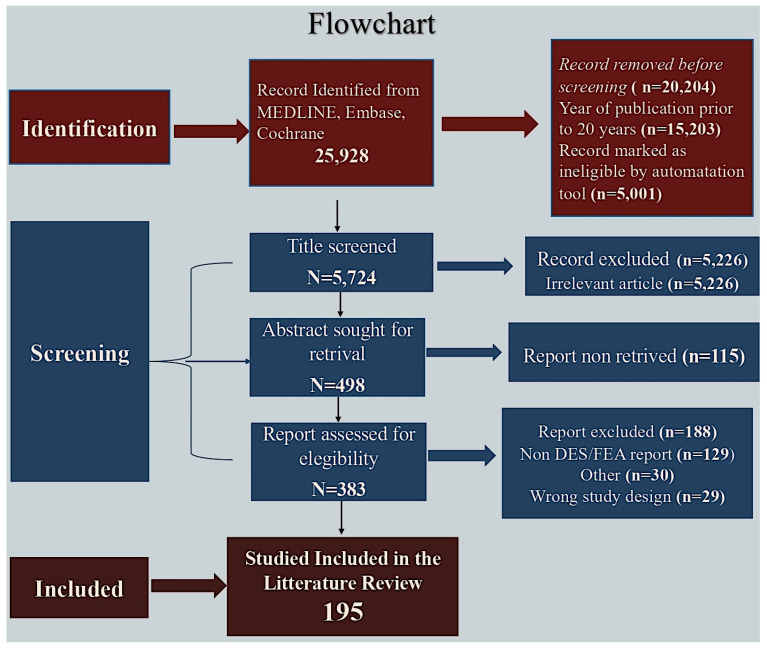
Flowchart.

The narrative review was designed, and the database investigation was conducted in July 2025 (Table 1). A comprehensive investigation was conducted using the following search terms in MEDLINE, Embase, and the Cochrane Library: drug-eluting stents; percutaneous coronary intervention; stent restenosis; ultrathin-strut DESs; computational modeling and FEA. The previously mentioned terms were searched for in combination with additional terms, including “STEMI,” “NSTEMI,” “crimping,” “deployment,” “recoil,” and “dogboning.” The publications selected for review focus on works from 2004 to 2025, with a particular emphasis on more recent literature. Additionally, a thorough search of the reference lists of articles identified by this search strategy was conducted, and relevant articles were selected. The search was focused on identifying data from the following sources: randomized controlled trials (RCTs), meta-analyses, and observational cohort studies. The collection of review articles was curated to offer readers more detailed background references and further reading material. Figure 1 presents the study’s flowchart.

## 2. Key Developments from Previous to Present-Day Coronary Stent Designs

Coronary stents have a remarkable history, as detailed in extensive reviews elsewhere [32,33], and will therefore only be briefly presented here. These implants have been used as vascular scaffolding in recent years. The terms “BMS,” “DES,” and “BRS” will be used to reference stents. Additionally, the terms “BRS” and “scaffold” will be used to refer to the application of scaffolding. The initial implementation of the BMS [34] was a critical milestone in the field of coronary artery disease intervention. However, the initial implementation of the BMS resulted in instances of in-stent restenosis (ISR) with rates up to 20%, necessitating revascularization procedures for a notable proportion of patients [35]. The success of these products can be attributed primarily to their minimalist concepts and notably their substantial struts. Almost a decade later, DES was introduced into clinical practice. This development marked a significant advance in the field, with the introduction of novel anti-proliferative drug coatings successfully reducing ISR from 17% to around 4% [36].

However, within 1–2 years of being implanted, first-generation DES led to a significant number of very late STs [37]. This has prompted a lively debate within the cardiology community, with some arguing that BMSs are comparatively less risky than first-generation DES due to their lower late thrombosis rates. The second generation of DES was introduced into the clinical setting around 2007, following several advances in the field. Cobalt, chromium, and platinum alloys were used to replace stainless-steel materials, allowing for the use of thinner struts (80–90 μm compared to 132–140 μm previously [38]). The result was minimized blood flow disruption surrounding the stent struts, enhanced biocompatible coatings, and reduced toxicity of anti-proliferative medications [39]. In recent years, the use of DES with ultrathin struts of less than 70 µm and bioresorbable polymer coatings has emerged as a promising solution, contributing to a significant reduction in ISR with a less than 3% requirement for revascularization (down from 4% previously) for newly formed coronary artery abnormalities, excluding bifurcations and chronic complete vessel obstructions [40]. It is encouraging to note that late ST has been effectively managed by reducing it from 2.5% to 0.6% [41]. However, the risk of early ST remains a concern, even for the latest generation of DESs, necessitating the use of dual antiplatelet therapy (DAPT) or blood thinners. The recommended treatment duration is currently under review. This review takes into account several factors, including the clinical benefits, such as minimizing DES-related early ST and bleeding complications; patient wellbeing; and the significant financial burden of continued treatment [42,43].

Contemporary DES frameworks are designed to meet the most rigorous standards in the field. They consist of three principal components: struts, crowns, and connectors. These elements are intricately linked, forming a robust structural framework. The rings are interconnected using purpose-designed connectors. The majority of commercially available DES currently in use feature an open-cell design, comprising rings and connectors of various shapes. The current industry standard for rectangular struts is a thickness between 74 and 90 μm. Two exceptions to this standard are the circular cross-section strut Resolute Onyx (Medtronic Vascular) and the 60 μm ultrathin-rutted Orsiro (BIOTRONIK SE & Co. KG, Woermannkehre 1, 12359 Berlin, Germany).

In a similar manner, the majority of stent rings are linked to one another via connectors. However, the double-helix Orsiro stent [44] and single-helix Resolute Onyx stent [45] are distinguished by their unique design, where the rings are preconnected through the helix. This feature is further enhanced by the S-type connectors (Orsiro) or spot welds between adjacent crowns (Resolute Onyx). The most prevalent DES alloy is cobalt–chromium (CoCr), and there are only two commercially available stents that diverge from this: the Synergy (Boston Scientific Corp. 300 Boston Scientific Way, Marlborough, MA 01752, USA) stent, which is composed of platinum–chromium (PtCr), and the Resolute Onyx stent, which has an inner platinum–iridium (PtIr) core encased in a CoCr shell. Regarding stenting, earlier studies indicated the use of paclitaxel-eluting stents [46] or sirolimus-eluting coatings [36]. Studies have demonstrated that sirolimus- or siro-limus analog-based coatings are often used due to their proven effectiveness in improving outcomes related to ISR and ST [47]. Torii and associates have reported further information on current stent configuration concerns and prospective future developments in DES [48]. In addition to stent configuration, the choice of base materials and manufacturing processes impacts the clinical efficacy of these devices, including the ISR and ST models. However, it should be noted that the topics in question fall outside the work’s current sphere of focus. Readers seeking more comprehensive information are directed to consult the other review articles [49,50,51].

A key concern in the field of clinical enhancement pertains to the long-term implications of durable electric suture (DES) applications, which have the potential to lead to long-term complications. These include aberrant vasomotor phenomena, the likelihood of late stent fracture, malapposition occurring between six and nine months post-implantation [52], and the persistent threat of stent malfunction [53].

In 2012, BRS was implemented at the clinic to address the identified issues. The rationale behind leveraging BRS is that it is only required during the remodeling phase following the implementation of a scaffold within the arteries. The scaffold prevents the arteries from becoming tight again and from narrowing again. In contrast to the BMS and DES scaffolds, which are composed of nonbiodegradable polymers or metals, these scaffolds are made of biodegradable polymers or metals. As the name indicates, they are designed to fully disintegrate within a period of two to three years. However, the initial BRSs were considerably thicker than DESs (approximately 150–170 μm) to counterbalance the reduced material strength [54], while maintaining scaffolding functionality. While BRS was regarded as an extremely promising development, the findings from longer-term trials in 2017 were disappointing. These results revealed significantly higher ST rates of up to 2.5% within three years (compared to 0.6% for the latest DES) [55], which led the product’s manufacturer, Abbott Vascular, to discontinue it in 2017. Consequently, the perceived risk associated with BRS has diminished. However, ongoing studies are addressing the outstanding issues with stents, the most widely used medical device to date [56,57,58].

Despite the initial challenges observed with the first generation of Absorb BRS (Abbott Vascular, Santa Clara, CA, USA), the development of new scaffolds is progressing well, with over twenty currently in active production. The objective is to address the identified shortcomings [58]. A new design has successfully reduced strut thickness for polymer-based scaffolds, making it applicable to both Poly-L-Lactic Acid (PLLA) and MeRes100 (Meril Life Sciences, Vapi, India). The strut thickness for PLLA has been reduced from approximately 150 μm to 95 μm for Arteriosorb (900 E. Hamilton Avenue, Suite 300 Campbell, CA 95008, USA), and the strut thickness for MeRes100 has been reduced from approximately 100 μm to 95 μm. Arterisorb [53] is an innovative stent that features a radial strength-enhancement strategy. This strategy is achieved by integrating advanced design features, including smaller cells at the stent center, and state-of-the-art manufacturing processes, such as melt processing and die drawing. This approach successfully achieved two key objectives: enhancing the radial strength while concurrently reducing the thickness of struts. This resulted in enhanced structural integrity and improved performance.

In addition to PLLA, a thin-strut BRS called the Fantom Encore (REVA Medical, San Diego, CA, USA) based on a proprietary polymer was released recently (2018). This product is manufactured by Thyrocare and has a strut thickness of 95–115 μm. Most polymer-based scaffold designs in development use metals owing to their inherently high material strength. However, a few use polymers instead. Two such examples are the magnesium-based BRS Magmaris (BIOTRONIK SE & Co. KG, Woermannkehre 1, 12359 Berlin, Germany) with a strut thickness of 150 μm [59] and the iron-based scaffold IBS (LifeTech Scientific Building, Keji 12th Road South, High-tech Industrial Park, Nanshan District, Shenzhen City, Guangdong Province, 518063, China) with a strut thickness of 70 μm. Both are currently undergoing human trials. Continued advances in thin-strut BRS innovations have the potential to transform the landscape of future stenting technologies.

Stents are medical devices used to prop open blood vessels that have undergone occlusion. These devices are utilized not only in coronary arteries, but also in a variety of other blood vessels. The following are included: the peripheral arteries (e.g., the femoral and iliac arteries [60]), ureters [61], and tracheal and bronchial airways [62]. The vast majority of these instances of obstruction are resolved through the deployment of self-expandable stents, crafted from a nickel–titanium alloy (Nitinol) that possesses exceptional elasticity. The stents under discussion differ from balloon-expandable coronary stents in several ways. These include differences in design, deployment methodology, deformation mechanism, and structural mechanics [60]. The core of our business is the development of coronary stent designs and their performance metrics. However, the optimization methodologies we present can be applied to a wide range of stents.

This report evaluates ten types of UtS-DES: those with a cross-profile of 0.96 microns or less and those with a cross-profile greater than 0.96 microns.

This review focuses on ten types of UtS-DES that have been evaluated. The types are categorized as follows: those with a cross-profile of 0.96 microns or less, and those with a cross-profile greater than 0.96 microns (Table 2).

### 2.1. UtS-DES with Very Low Crossing Profile

♦ Coroflex ISAR Neo

**Figure 2:**
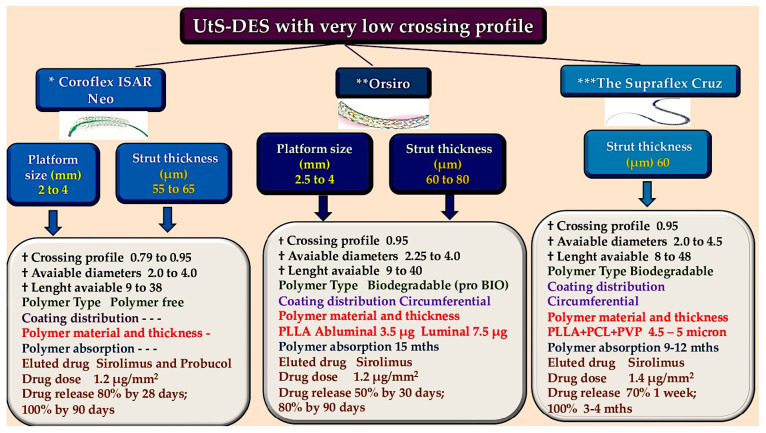
Current characteristics of UtS-DES with very low crossing profile. Months, mths; PCL, poly-caprolactone; PLGA, poly-D, L-lactide-co-glycolide; PLLA, poly-L-lactic acid; proBIO, amorphous hydrogen-rich silicon carbide; PVP, poly vinyl pyrrolidone. The thickness of the strut is manufactured using cobalt chrome. * B.Braun, Melsungen, Germany; ** Biotronik Bülach, Switzerland; *** Sahajanand Medical Technologies, Surat, India; † mm (crossing profile, avaiable diameters and length).

The Coroflex ISAR stent (B. Braun, Melsungen, Germany) is a stent composed of a polymer-free cobalt–chromium alloy, to which sirolimus is added as an eluting agent. The stent platform’s dimensions have been developed based on two stents: the CX-Blue Ultra stent, suitable for diameters between 2.0 and 3.0 mm (55 mm), and the CX-Blue Neo stent, suitable for diameters between 3.5 and 4.0 mm (65 mm) (Figure 2). The innermost layer of the stent—devoid of polymers—is positioned on the interior surface of the microporous structure. The formulation consists of sirolimus at a concentration of 1.2 µg/mm^2^, in conjunction with probucol, to regulate the delivery of the administered medication [68].

Probucol has been demonstrated to function as a matrix builder. The drug has been found to be a highly lipophilic agent with lipid-lowering properties and antioxidant characteristics. It has been determined that 80% of sirolimus is released within 13 days, with the remaining 20% released over the course of 19 days. The device is manufactured in diameters ranging from 2.0 to 4.0 mm and lengths between 9 and 38 mm. Massemberg et al. [69] reported the findings on the utilization of Coroflex intracoronary stenting and its angiographic outcomes. The present study constitutes a non-inferiority trial, with a sample size of 3002 patients. The objective of the study is to make a comparison between the efficacy of sirolimus- and probucol–zotarolimus-eluting stents (ISAR-TEST-5).

As demonstrated in the primary endpoint analysis, which included a combination of cardiac mortality, target-vessel-related MI (TV-MI), or target lesion revascularization (TLR), the Coroflex ISAR stent showed non-inferiority to the ResoluteZES (P-non-inferiority = 0.006; P-superiority = 0.74) [69]. The validity of these findings was confirmed at the 5- and 10-year follow-ups, which revealed a low incidence of probable or definitive ST in both cohorts. The findings were consistent across predefined groups based on age, gender, diabetes status, and artery dimensions [70,71].

♦ Orsiro

The Orsiro coronary stent (BIOTRONIK, Bülach, Switzerland) consists of an UtS, a cobalt–chromium alloy, and a sirolimus-eluting polymer that is bioresorbable (i.e., biodegradable) (biodegradable polymer sirolimus-eluting stent (BP-SES)). The product’s dimensions range from 2.25 to 4.0 mm in diameter and from 9 to 40 mm in length. The diameters of the stents range from 2.25 mm to 3.0 mm, with strut thicknesses measuring at 60 micrometers. In contrast, devices with a diameter between 3.5 and 4.0 mm have strut thicknesses of 80 micrometers [28].

The Orsiro BP-SES has a layered composition. The internal layer consists of a cobalt–chromium alloy (PRO-Kinetic Energy™ stent) configured in a dual-helix configuration, a design conceived to enhance delivery by reducing the crossing configuration. The proBIO polymer layer prevents direct contact between the metallic stent platform and the blood vessel or circulatory system (Figure 2).

According to the available evidence, the aforementioned coating has been identified as an amorphous hydrogen-rich silicon carbide coating adhered to a metallic substrate. The proBIO coating is a distinctive attribute of the stent that has the potential to exert favorable effects, including a reduced rate of metallic stent deterioration and diminished tissue inflammatory responses, encompassing allergic responses to the metallic constituents. The coating’s “passive shield” is of particular interest, as it is characterized by its permanence. That is, the coating does not delaminate over time. The external surface is composed of a bioabsorbable poly-L-lactic acid (PLLA) polymer loaded with the pharmaceutical substance sirolimus. The BIOlute™ coating features a specialized distribution pattern, with a thickness of 7.5 µm on the inner surface and a reduced layer measuring 3.5 µm on the luminal part of the stent. The sirolimus dosage was set at 1.4 mg/mm^2^. Over a period of two years, there is an observed degradation process of the PLLA. This results in the release of 50% of the drug substance within a 30-day timeframe, as well as 80% of the substance during the initial three months. A thorough evaluation of the safety and effectiveness of the Orsiro SES was carried out through a series of RCTs in different settings. These included acute and chronic coronary syndromes, along with various types of lesions, such as de novo lesions, small vessel disorder, chronic complete obstructions, and in-stent restenosis [28].

♦ The Supraflex Family

The Supraflex system, manufactured by Sahajanand Medical Technologies in Surat, India, consists of an UtS stent measuring 60 μm. The device is fabricated from a Co-Cr L605 alloy and a biodegradable sirolimus-releasing polymer. The range of stent sizes suitable for use in this procedure varies from 2.00 mm to 4.50 mm, with lengths ranging from 8.00 mm to 48.00 mm. The sirolimus-based solution has a target dosage of 1.4 milligrams per square centimeter. Its polymer formulation is designed to degrade gradually over a period of 9 to 12 months. The drug is released within seven days. The latest version of the Supraflex, known as the Supraflex Cruz, incorporates dual-Z connectors that extend the full length of the struts from “valley to valley.” The objective of this design enhancement is to enhance delivery capability and augment the flexibility of the stent. Additionally, the stent features an advanced probe section that has been redesigned to improve pushover capability.

A number of ongoing investigations will provide the necessary data to assess the performance of Supraflex Cruz SES in various contexts, including ACSs, multivessel disease, and individuals with a high bleeding risk (HBR) [72,73]. TALENT, a multivessel trial, aims to provide a comparative analysis of clinical outcomes observed in a sample group of 1550 patients diagnosed with three-vessel disease. This comparative analysis will help evaluate the effectiveness of different treatment options for patients with three-vessel disease. The study will include a thorough evaluation of the SUPRAFLEX Cruz SES and the SYNERGY SES. The database is designed to accommodate a total of 1200 participants [74] (Figure 2).

### 2.2. UtS-DES with Low Crossing Profile

♦ Mi Stent

**Figure 3:**
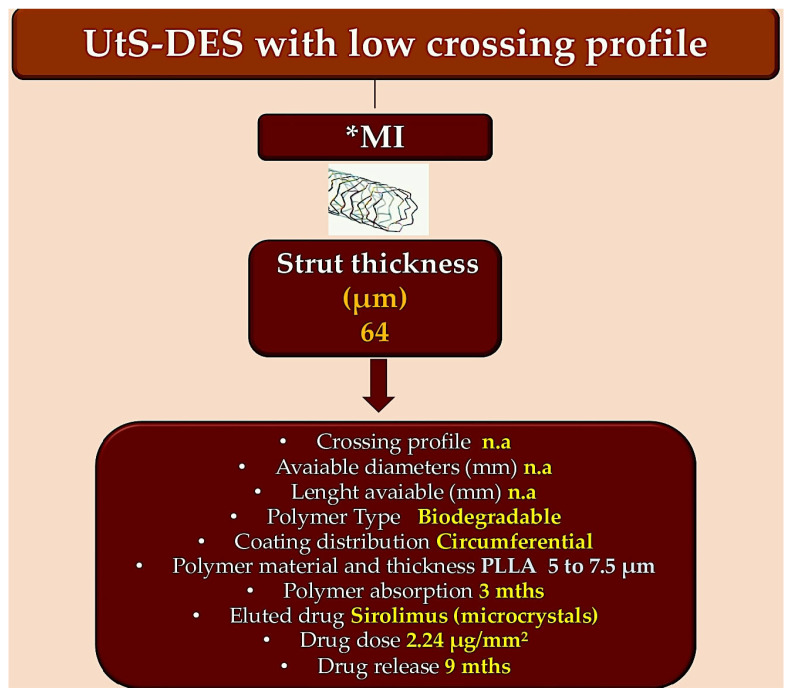
Current characteristics of UtS-DES with low crossing profile MiStent SES. mths, months; n.a, not applicable; PLLA, poly-L-lactic acid. The thickness of the strut is manufactured using cobalt chrome. * MiCell Technologies, based in Durham, NC, USA. MIstent Elixir Medical.

The MiStent Sirolimus-Eluting Stent (MiStent SES) is a medical device developed by MiCell Technologies, a company based in Durham, Nord Carolina (NC), USA. The stent is 64 mm in size and is made of a biodegradable polymer sirolimus-eluting stent. The primary use of the stent is in the treatment of cardiovascular conditions in patients. Sirolimus is synthesized inside the vessel wall as microcrystals. Its crystalline structure enables controlled release of the drug. Figure 3.

It is noteworthy that the polymer, PLLA, is gradually eliminated from the body approximately three months following implantation, thereby reducing the risk of vascular inflammatory response. Conversely, sirolimus is administered with continuous delivery for a duration of up to 270 days. The Mistent has undergone a thorough assessment within the Dessolve RCTs [75]. The most recent randomized controlled trial (RCT), designated as Dessolve III, demonstrated that the MiStent SES was non-inferior to the Xience EES. This pertained to the primary endpoint of cardiac death, target-vessel MI, or clinically indicated target lesion revascularization at one year. The current trial involved a total of 1398 patients from twenty different European medical centers.

♦ Biomime, Biomime Morph, and Evermine 50

BioMime (Meril Life Sciences Pvt. Ltd., Vapi, Gujarat, India) is a cutting-edge stent manufactured with a thin (65 μm) cobalt–chromium biodegradable polymer coated with sirolimus [76,77,78]. The features of this ultrathin-strut stent are notable for their hybrid design, incorporating sealed cells at either end and open cells in the middle. This configuration is intended to enhance stent expandability and reduce the risk of edge dissection. The mid-part of the stent features an open-cell design, allowing for convenient access to and treatment of side branches. The biopolybiodegradable polymer currently under consideration has a minimal thickness of approximately 2 µm. The product is composed of PLLA and poly-D, L-lactide-co-glycolide (PLGA). The degradation timeframe of the polymer is approximately 60 days.

**Figure 4:**
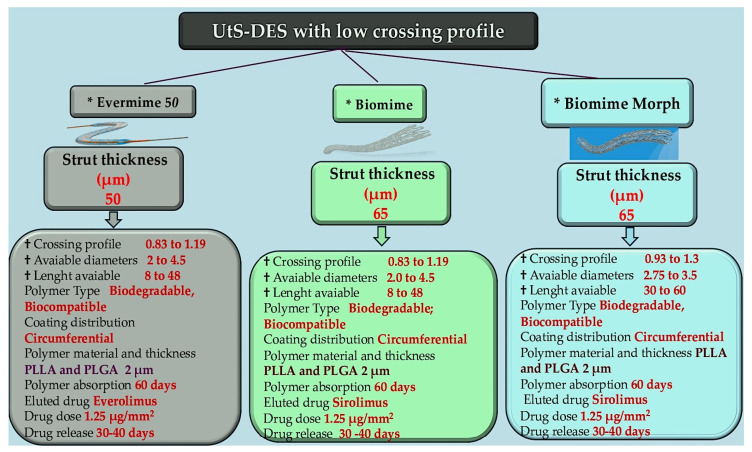
Current characteristics of UtS-DES with low crossing profile; PLGA: poly-D, L-lactide-co-glycolide; PLLA: poly-L-lactic acid. The thickness of the strut is manufactured using cobalt chrome. * Meril Life Sciences Pvt. Ltd., Vapi, Gujarat, India; † mm (crossing profile, avaiable diameters and length).

Furthermore, the sirolimus release profile, measured at 1.25 µg/mm^2^, is initiated 30 to 40 days post-stent implantation. The BioMime SES is available in lengths ranging from 8 to 48 mm and diameters from 2.00 to 4.50 mm (Figure 4).

The RCTs (i.e., MeriT-1, MeriT-2, and MeriT-3) have demonstrated the safety and effectiveness of the biomimetic SES (i.e., a device that mimics the behavior of a natural body tissue) for the management of both de novo coronary lesions and those of a more complicated nature [76,77]. The MERI-V trial was the first to compare the Biomimex SES with the Xience EES in a 2:1 ratio among 256 individuals [78]. The meriT-3 RCT was a substantial project that engaged 1161 participants from diverse demographic backgrounds. The subjects of this investigation underwent PCI with Biomime SES across 15 centers in India.

The Biomime Morph (Meril Life Sciences Pvt. Ltd., Vapi, Gujarat, India) represents a subsequent development of the Biomime SES innovation. This stent incorporates a sophisticated tapered configuration with two proximate and distal diameters (e.g., 2.75–2.25 mm; 3.00–2.50 mm, etc.). The tapered stent system, available in lengths of 30, 40, 50, and 60 mm, is an optimal solution for the management of extensive, elongated lesions.

The Evermine 50 (Meril Life Sciences Pvt. Ltd., Vapi, Gujarat, India) employs the same hybrid cell stent concept but features a Co-Cr L605 alloy that is 50 µm thinner. The stent is engineered to release everolimus at a controlled rate of 1.25 milligrams per square millimeter, ensuring precise and predictable drug delivery.

### 2.3. Auxetic UtS-DES

**Figure 5:**
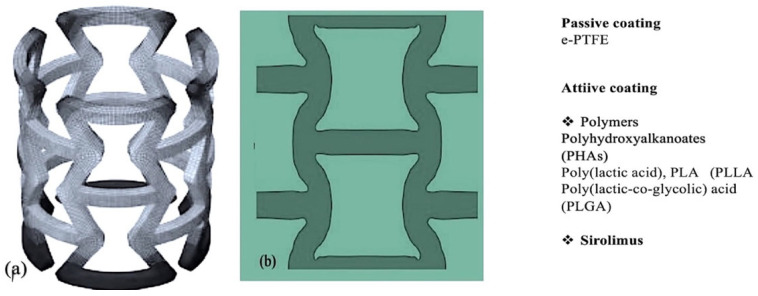
(**a**) The bioresorbable UtS-DES System is coated with a film of e-PTFE. (**b**) The UtS-DES model incorporates sirolimus and biodegradable polymers (PLLA or PLGA or PHAs).

The auxetic UtS-DES system has the potential to represent a significant breakthrough in the field of interventional cardiology. It is designed to provide a customizable solution that is tailored to patients’ anatomical and pathological characteristics. The product’s ultrathin struts and auxetic properties have been developed to ensure it adapts to the varying angles of the secondary coronary branches as well as to variations in size of the main coronary branch along a maximum extension of 50 mm [79,80,81,82,83,84,85,86,87] (Table 3 and Figure 5).

The auxetic UtS-DES System is coated with a film of expanded politetrafluoroethylene (e-PTFE). This novel combination of materials is a pivotal innovation that offers the distinct advantage of a reduced risk of inflammatory and thrombogenic reactions. For the auxetic UtS-DES model, the solution that has been selected is a pioneering dual approach that incorporates sirolimus and biodegradable polymers such as PLLA, PLGA, or polyhydroxyalkanoates (PHAs). The purpose of this combination is to create an auxetic UtS-DES device. The selection of biopolymers is based on the characteristics inherent to each particular lesion. The resorption time varies from a minimum of four to a maximum of 24 months, depending on the lesion’s properties.

PHA is a highly biocompatible and environmentally friendly substance, making it a suitable component for use in elastic medical devices in the field of biomedical engineering [88]. In the current landscape of PHAs, 4-hydroxybutyrate (4HB) polymers represent a unique class with known clinical applications. These are primarily used in sutures and cardiovascular stents [89]. A rise in the 4HB fraction results in greater extensibility of poly(3-hydroxybutyrate-co-4-hydroxybutyrate) (P(3HB-co-4HB)), making it a promising material for use in extensible medical devices [90,91].

The following is a detailed description of the advanced manufacturing process for the auxetic UtS-DES System. The development of customized cardiac stents for personalized medicine is rapidly progressing, driven by the convergence of advanced microfabrication, precision laser engineering, and hybrid material processing technologies. Stent fabrication technologies include laser cutting, micro-EDM, additive manufacturing, and shape memory alloy machining. However, femtosecond laser ablation is a technology that stands out due to its remarkable precision, minimal thermal damage, and its ability to generate complex geometries, which are essential for patient-specific implants. The envisioned workflow entails the micro-machining of a thin roll of nitinol using a custom femtosecond laser setup (OPTOPRIM SAS 40 91300 Massy France ) equipped with a Laser Carbide source from Light Conversion (Vilniaus, Lithuania). This innovative system integrates a beam expander and a 10× lens, offering a 10 mm focal distance and enabling the production of tightly focused ultrafast pulses. The nitinol roll is mounted on a fully programmable 5-axis stage driven through a Python interface. This allows for dynamic, high-accuracy shaping of intricate stent architectures, which can be tailored to individual cardiovascular anatomies. Following the laser structuring process, a coating of expanded polyterhafluoroethylene-based polymer is applied by rotational spin-coating. This establishes a low-friction interface that is biocompatible. Subsequent gas-foaming transforms the coating into a porous structure that is comparable to e-PTFE. This process enhances tissue integration and hemocompatibility, making it a valuable asset for medical applications. Additionally, the process has the capacity to incorporate an optional layer of sirolimus in conjunction with PLA, PLGA, or PHAs. This deposit is in the form of a thin film measuring from 4 µm to 15 µm of BP, along with a sirolimus concentration ranging from 1.4 µg/mm^2^ to 2.4 µg/mm^2^ (microcrystals). This opens up the possibility for biodegradable or drug-eluting functionalities. This integrated method establishes the use of femtosecond laser technology for nitinol processing. It is a significant step in creating next-generation, patient-specific cardiac stents.

## 3. Computational Model for Native Coronary Artery

### 3.1. Definition of a Computational Atlas of Normal Coronary Artery Anatomy

A number of studies have used procedural cineangiograms [14,92,93,94] or coronary computed tomography angiograms (CCTAs) [6,95,96] to evaluate anatomical alterations in coronary artery angles, bifurcations, and diameter. The imaging was performed using a multidetector CT scanner with retrospective ECG gating. The procedure was initiated following the administration of 60–80 milliliters of Omnipaque 350 contrast medium. Beta blockers were prescribed to achieve and maintain a target resting heart rate of approximately 60 beats per minute. Prior to undergoing imaging procedures, the patient received sublingual nitroglycerin spray. The end-diastolic CT image data from 75% of the heart’s cycle have been converted into a 3D model consisting of about 200 images captured from the transverse plane and assembled [78].

In our experience, pre-operative CCTA examinations were performed on patients with CAD using a dual-source computed tomography scanner (GE Revolution™ 160; GE Healthcare, CS 20529 78457 Vélizy, France). The production of contrast-enhanced images is contingent upon the injection of an iodinated contrast agent. The primary scan parameters for CCTA include the following: mixed axial, gated and helical ungated; 160 kV; Smart mA; Dose-length product (DLP) 475 mGy.cm; body mass index (BMI); 60–96 beats per minute (bpm) [1,2,3,97].

The CCTA data sets were processed using ITK-SNAP version 2.4 [1,2,3,98,99]. Specifically, a region of interest is delineated in relation to the reconstruction as a whole by leveraging the software’s functionalities for contrast, cropping, and segmentation. The region in question comprises the aortic root, the coronary ostia, and the coronary branch. It is crucial to closely examine the bifurcations and alterations in the caliber of the primary coronary branches. The use of a variety of Hounsfield unit thresholds is an effective method of distinguishing the calcific component of the aortic root and coronary artery from the surrounding healthy tissue.

This methodology enables the execution of precise evaluations with regard to both location and dimension. Once the segmented region has been extracted, the aortic and coronary lumens, in conjunction with the calcium deposits, are exported in the form of stereolithographic (STL) files. The native aortic root model is of the highest significance because the coronary ostia are an integral part of the right and left coronary sinuses, which are located within the aortic root. The dynamics of flow into the coronary arteries occur during the diastolic phase, with these phenomena being determined by the mechanics and function of the aortic root.

- Segmentation

A standard analysis pipeline will be developed to ensure consistent processing of all instances with minimal user interface interaction. A seasoned analyst has performed the segmentation procedure using a semi-automatic method that has previously been validated. This method is estimated to require 20 min per study [4,5,6,7,8,9].

Segmentation pipeline outputs and typical results will be saved in image format. As per standard protocol, it is essential that all meshes be subject to visual inspection in order to ascertain that they meet the required quality standards. In the event that a mesh is found to be of substandard quality, it will be excluded from the atlas. The number of mesh exclusions will be clearly indicated. Please be advised that MATLAB Release 2025b (The MathWorks, Inc., Natick, MA, USA) [10] will be employed for the purpose of computing all vessel measurement data.

- Labeling

The manual assignment of a label to each centerline is as follows: left anterior descending (LAD), diagonal (D1), septal, left circumflex (LCX), obtuse marginal (OM1), intermediate, right coronary artery (RCA), and acute marginal. It is crucial to note that other central lines were not addressed. A study was conducted to determine the location of the crux [26]. The crux is defined as the area on the posterior aspect of the heart where the coronary sulcus (the groove separating the atria from the ventricles) and the posterior interventricular sulcus (the groove separating the left from the right ventricle) intersect. At this stage, the blood vessels are divided into separate branches. This is a key point of distinction in patients with right-dominant circulations. As demonstrated in studies [11,12,13,14,15], the bifurcation giving rise to the posterior descending and posterolateral branches was located in the study subjects. In the event of multiple vessels being present, please note that only the first branch will be analyzed, unless more than one vessel is documented. In instances where the same description is applied to multiple vessels, such as in cases of diagonally oriented vessels, only the initial branch will be analyzed.

- Definition and Computation of Angles

The system is designed to automatically detect the point at which the central lines divide and calculate a bifurcation point. The following observations were made for measurement purposes. The measurements were taken at a range of depths from the specified bifurcation point. The measurement process is carried out in accordance with the centrelines, and each incremental step is taken in increments of 0.5 mm. At each stage of the process, a bifurcation plane was delineated (a least-square plane fitted to all of the centerline points from the bifurcation plane to the specified depth).

At each bifurcation, four angles were analyzed in 3D: the inflow angle (the angle at which the proximal vessel enters the bifurcation plane) and the bifurcation angle (the angle of the bifurcation between the distal main vessel and the side branch). Furthermore, the angles between the main vessel and the side branch, as well as those between the main vessel and its distal end, must be measured precisely. The determination will be formally represented using dedicated figures. To facilitate comparison, we will also consider the angle B’ measurement, which is obtained by discounting the curvature of the distal main vessel [11,12,13,14,15]. From a computational standpoint, it is essential to employ linear approximations of the central lines to ascertain these angles.

The figures obtained with precision showed the bifurcation angle as a function of distance from the bifurcation point (or depth). Angle measurements are defined as the arithmetic mean of the values obtained at a depth interval of 5–10 mm, where variations are generally minimal. It is essential to acknowledge the incorporation of a sign convention pertaining to the inflow angle in relation to the center of the heart. In instances where the bifurcation aligns with the expected convex curvature of the heart, a positive sign will be employed, while a negative sign will be used when the alignment differs from the anticipated curvature [11,12,13,14,15].

- Computation of Diameters

The accuracy of measurement was ensured by determining the diameter of each vessel at 0.5 mm intervals. This process started at the ostium, or the previous main bifurcation, and continued to the final available point. At each intersection, the mesh is intersected with a plane normal to the centerline, which results in a polygon shape. The area was determined by calculating the polygon’s shape. The effective radius (R) and diameter will be calculated from the specified area (A) using the following formula: A = πR^2^. At a bifurcation, there is a short-term increase in the effective radius within the confluence polygon [16,17]. In this region, the area was maximized at the bifurcation point. The ellipse was fitted in this instance, with two diameters derived from the major and minor axes. The objective of this analysis was to facilitate the execution of relevant calculations, including those pertaining to eccentricity and coronary flow [18,19,20,100].

### 3.2. Finite Element Analysis Model

#### 3.2.1. Native Aortic Root

The configuration of the native aortic root is as follows: The STL file of the aortic root will be obtained through processing in Matlab (The Mathworks Inc., Natick, MA, USA), a method that allows the definition of a set of splines, which resemble the cross-sectional contours of the aortic root. These curves will be used to automatically generate a volume model of the aortic root wall. The next step is to import the data into ABAQUS CAE software (Simulia, Dassault Systems, Providence, RI, USA) to set up the finite element analysis. The geometrical model of the aortic root and coronary artery branch distribution, which was derived from the STL file, serves as the foundation for the finite element analysis of PCI procedures in an animal model. It is important to note that our procedure generates both the aortic wall and the native valve leaflets. This facilitated the acquisition of a thorough and accurate simulation model. To incorporate the geometry of the native leaflets and their distance from the coronary ostia, it was necessary to identify nine reference points.

The reference points were essential in determining the precise planes. These planes subsequently furnish the partition with data regarding the entire aortic root model and coronary ostia location. This innovative technique allows for precise extraction of the leaflet’s commissures and attachment lines, ensuring a meticulous approach to cardiac procedures. To accurately determine the length of the free margin, ultrasonic measurement was performed in an in vivo model. The delineation of the margin was performed using the circular arc method, a technique that has proven to be highly effective in such applications. After establishing the leaflets’ perimeter, the subsequent step involves reconstructing their surface in the open configuration and determining their relationship with the coronary ostia that receive blood flow during diastole.

The model of the aortic wall was divided into tetrahedral elements, with the healthy region and the calcified part modeled distinctly. The leaflets were segmented using shell elements with reduced integration for healthy tissue and for the calcified region.

For the purposes of this analysis, it has been assumed that the material responsible for the problems in the native aorta is isotropic and homogeneous. This finding aligns with the research conducted by Gnyaneshwar et al. [101] and Capelli et al. [102]. The most important aspect of reproducing material behavior is the use of reduced polynomial forms. This strategy has proven to be highly effective [103,104,105,106,107,108]. The calculation of the reduced polynomial strain was conducted along with the reporting of the constant values that resulted from the implemented fitting procedure, as outlined by Auricchio et al. [105]. It is assumed that the aortic wall and the native valve leaflets have uniform thicknesses of 2.5 and 0.5 mm, respectively. According to the research by Capelli et al. [102], the defining characteristics of calcified tissue include an elastic modulus of 10 MPa, a Poisson ratio of 0.35, and a density of 2000 kg/m3. The thickness of the calcified shell elements will be set to 1.4 mm.

#### 3.2.2. Coronary Artery

- Tensile stress at the elbow point

The mechanical properties of coronary arteries were obtained using cadaver samples of non-pathological tissue (cardiovascular diseases). Maximum lengths of more than 50 mm were recorded [108].

In order to assess the health of a patient’s coronary arteries, it is essential to determine the relationship between actual stress (σ) and strain (λ). This relationship was determined through experimental load–displacement curves. As detailed in the various studies, the value of elongation (λ) was determined by dividing the measured length (l) by the initial length (L) of the specimen [109,110,111]. The value of calculated stress (σ) is derived by dividing the applied load (P) by the cross-sectional area (a). The assumption of incompressibility was used to calculate the relationship between the current (A) and initial (A_0_) cross-sectional area, expressed as A = A/λ.

For low levels of applied stress, the aortic tissue displays a high degree of compliance. However, when stresses exceed the threshold, the tissue demonstrates a significantly stiffer response. To summarize the main mechanical response of the arterial wall, the stress-stretch curve was condensed into three parameters. In our analysis, we consider the stretch and stress at the breaking point (σ_R, λ_R), as well as the stress at the transition point, or “elbow,” between the compliant and the stiff regions (σ_e). The breaking point of the sample (σR, λR) was defined as the first point at which one of its layers failed, an occurrence that could be easily identified in the tensile curve by a sudden drop in the load. The elbow stress (σ_e) was calculated using the change in the first derivative of stress with respect to stretch.

As demonstrated by the findings of Claes et al. [110], the variation in elbow stresses is contingent on age. As people age, their elbow joints tend to experience less stress. In younger individuals, the area of the heart affected by blood flow is typically located below the level of elbow stress. The elbow stresses are nearly equivalent to the working stresses of the artery for the remaining elements. These observations align with the findings of in vivo studies of aortic distensibility, which demonstrate a decline in arterial compliance with age [111]. The data regarding the wall distensibility is crucial in selecting the optimal bypass graft, aiming to minimize mechanical mismatch and thereby reduce stresses experienced at the anastomosis.

- Coronary wall strength

As outlined by Claes et al. in their study [110], the findings on the mechanical strength of human coronary arteries are among the inaugural results to consider the entire arterial wall. The data on circumferential tensile strength (σ_R) was presented as a function of age for coronary wall specimens. The only work addressing wall strength in coronaries is the study by Holzapfel et al. [112], which performed tensile tests on separated intima, media, and adventitia layers of coronary arteries from 13 patients aged 72 ± 7 years. As indicated in the study by Claes et al. [110], a strong relationship was found between the breaking conditions of the media layer and the key parameters discussed. The results indicate that the circumferential tensile strength recorded was 0.39 ± 0.07 MPa. This corresponds to the 0.45 ± 0.19 MPa reported in the aforementioned study. The absence of experimental data could result from the complexity inherent in obtaining coronary vessels. Their integration within the heart wall necessitates a specialized dissection procedure, and their mechanical testing requires a small dimension, which could be contributing to the issue.

When evaluated against other arteries, the values reported here for the circumferential strength of coronary arteries are significantly lower than those published for the ascending aorta. It is important to note that both the coronary arteries and the ascending aorta exhibit the same elastic properties due to their proximity. As detailed in the report by Vorp et al. [113], the circumferential strength of the ascending aorta in donors aged 51 ± 6 years was found to be 1.80 ± 0.24 MPa. In a separate study, Iliopoulos et al. [114] reported a mean strength of 1.63 ± 4 MPa in a group aged 66 ± 3 years. These values are clearly above the 0.39 ± 0.07 MPa benchmark set for coronary arteries of a similar age.

Regarding the impact of age, the circumferential tensile strength of the coronary arterial wall typically experiences a decline, which often occurs abruptly around the ages of 30 to 40. This finding aligns with the published research on the mechanical properties of other arteries, such as the ascending aorta [115] and the descending aorta [116,117]. In many cases, the decisions regarding the level of balloon inflation pressure and the size of the balloon during an angioplasty procedure have not taken into account the mechanical properties of the coronary artery being treated. However, it should be noted that the inflation of balloons at high pressures has the potential to result in substantial damage to the arterial wall, including dissection and the formation of an occlusive intimal flap. The findings outlined by Claes et al. [110] have enabled the determination of the maximum acceptable levels of pressure and dilation for coronary arteries during transluminal angioplasty, considering the actual arterial strength. The application of pressure from the balloon can affect the arterial wall and atheroma plaque in the surrounding area, depending on their respective levels of stiffness. Consequently, cardiologists would find it advantageous to possess a comprehensive understanding of the mechanical characteristics of the arterial wall, which would be further enhanced by the incorporation of numerical simulations of angioplasties. This would allow them to ascertain the maximum inflation pressure of the balloon without risking damage to the artery.

- Governing equations and blood rheology

Blood flow in the coronary arteries will be assumed to be incompressible and laminar, which is appropriate for coronary Reynolds numbers and vessel diameters in the studied range. The flow will be governed by the unsteady incompressible Navier–Stokes equations:

- Continuity:
  ∇⋅u=0
- Momentum:
  $$\rho (\frac{\partial u}{\partial t}+u\cdot \nabla u)=-\nabla p+\mu \nabla^{2}u$$

where u is the velocity vector, *p* is the pressure, *ρ* is the density, and *μ* is the dynamic viscosity.

For the baseline simulations, blood will be modeled as a Newtonian fluid with:

- Density $\rho =1060\;{\mathrm{kg}/m}^{3}$;
- Viscosity $\mu =3.5\times 10^{-3}\;\mathrm{Pa}\backslash \mathrm{cdotps}$.

In certain instances, a non-Newtonian Carreau–Yasuda model is employed to assess the sensitivity of results to shear-thinning behavior, particularly in regions of extremely low shear. The corresponding parameters will be obtained from the literature and implemented via a user-defined viscosity law in the CFD solver.

## 4. Finite Element Modeling of Conventional DES and Ultrathin Struts DESs

The large proportion of DESs deployed thus far have incorporated non-degradable polymer coatings with anti-proliferative drugs to overcome the well-known issue of in-stent restenosis (ISR), which is an undesirable complication of bare-metal stents [24]. While DESs have been shown to reduce ISR rates by 74% in high-risk patients, concerns have been raised about the long-term implications of metallic-alloy stents under strain and under corrosive conditions. These stents could potentially lead to strut fracture and failure over time. It should be noted that this may result in potential complications, including delayed healing, lethal migration and unstable angina [107,108]. Bioresorbable stents represent a significant breakthrough in addressing the potential long-term complications associated with metallic stents. Biodegradable polymers currently represent the material of greatest interest in this field [118,119,120,121]. Fully expandable polymeric biodegradable stents are designed to provide initial structural integrity to the vessel wall as scaffolding, ensuring sufficient radial strength to counteract mechanical recoil following implantation. Arterial remodeling enters a stable phase within approximately six months, at which point no significant scaffolding is necessary.

Stents should ideally be designed to disintegrate within six months, leaving the vessel intact and free of any potentially inflammatory agents or impediments that could hinder subsequent treatment options [122]. As the process progresses, the polymers progressively become more malleable, enabling the dissipation of high stresses caused by the permanent stents within the artery undergoing recovery. Recent reports have indicated an apparent association between bioresorbable stents and incidents of stent thrombosis [123]. However, further investigation has revealed that the 2.5 mm platforms are linked to a higher frequency of such complications [124].

One of the primary concerns regarding polymeric BRS pertains to their performance under stress, particularly their interaction with blood vessels during and after deployment. Agrawal et al. [125] were the first to assess the in vitro performance of Duke’s biodegradable stents (MiStent SES, Durham, NC, USA), which are made of poly-L-lactide acid (PLLA) fibers. A study of the results revealed that the optimum combination of the mechanical characteristics of PLLA fibers and the stent geometry was essential to the successful development of a biodegradable stent. As stated in study [126], a woven fiber polymeric braided stent was tested in a laboratory setting using a process of radial compression. The performance of these stents was not as effective as that of metal ones. Additionally, the failure pressure was determined to be reduced, even for thicker PLLA fiber materials. Another study drew parallels between the in vivo efficacy of stents manufactured using biodegradable PLLA and stainless steel [127]. This objective was accomplished by observing the stents post-implantation in living pigs. The procedure was successful, with both stents being properly implanted. However, PLLA has been observed to be comparatively pliable when compared to metallic alloys. This has a bearing on the radial stiffness of the implanted devices, and further investigation is required [127].

Over the past two decades, significant progress has been made in the field of bioresorbable stent innovation. Numerous devices have undergone rigorous evaluations in both preclinical and clinical settings. MiStent SES-Elixir Medical Corporation, USA is a stent manufactured from PLLA that is among the most prominent biodegradable stent options currently being offered on the market. Radial strength of the polymer stents has been shown to be sufficient six months after deployment. Bioresorption occurred within the expected timeframe of one to two years after implantation [128]. FEAs have been instrumental in the development of models of stent expansion and deformation during deployment. However, it is important to note that the majority of these analyses were performed on metallic stents [129,130,131]. For instance, Imani et al. [132] developed a model to assess the impact of design on vessel wall stresses for Palmaz-Schatz, Xience V, and NIR stents. A comparison analysis revealed that the Palmaz-Schatz stent induced 15.6% and 7.6% higher levels of stress in the arterial wall compared to the Xience V stent and the NIR stent, respectively. Preliminary research indicates a direct correlation between vessel wall stresses and the occurrence of in-stent restenosis. The Palmaz-Schatz stent, which demonstrated the highest incidence of restenosis in clinical trials, is a good example of this correlation.

This claim is further supported by recent FEA simulations of metallic stents. The results of the simulations demonstrate that the configuration of the stent has a significant impact on the device’s deployment and the resulting stresses it generates within the arterial environment. FE modeling can also be used to optimize the shape of metallic stents, enhancing their resistance to fatigue and radial flexibility [133,134]. However, there is currently a lack of research addressing the deformation of bioresorbable polymer stents, particularly during their implantation in atherosclerotic vessels.

### Finite Element Modeling of Conventional DES and UtS-DES with Low Crossing Profile

The predominant view in the field is that the arterial layers demonstrate anisotropic behavior due to the reinforcement of two types of collagen fiber. In the context of finite element analysis, the Holzapfel–Gasser–Ogden (HGO) anisotropic hyperelastic model [112,135,136] is widely accepted and can be used to elucidate the anisotropic behavior of individual coronary arterial layers during the crimping stage and deployment (Table 4).

The calculation of hyperelastic strain energy potential, W, is determined by the following formula: [136]

*W* = *C*_10_(*I*_1_
*−* 3) + (*k*_1_/2*k*_2_) [exp(*k*_2_*<E_f_>*^2^) − 1] + (1/*D*)[(*J*^2^ − 1)/2 − ln*J*]

*E_f_* = k(*I*_1_
*−* 3) + (1 − 3_k_)(*I*_4_
*−* 1)

**Figure 6:**
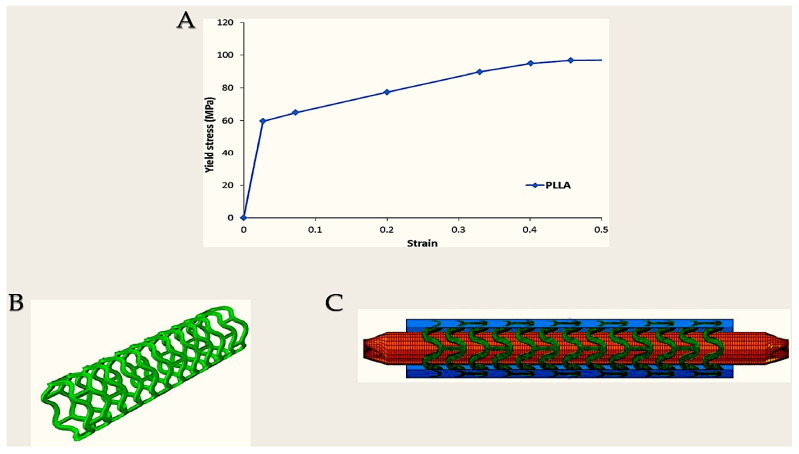
The biomechanics of the permanent implantation of the MiStent device, developed by SES-Elixir Medical Corporation of the USA, are outlined herein. (**A**) Stress–strain behavior for PLLA used in simulations. y, yeld stress MPa; x, strain %. (**B**) 3D models. (**C**) Procedure of stent crimping [120]. This article is distributed under the terms of the Creative Commons Attribution 4.0 International License (http://creativecommons.org/licenses/by/4.0/ 19 July 2026), which permits unrestricted use, distribution, and reproduction in any medium, provided you give appropriate credit to the original author(s) and the source, as well as a link to the Creative Commons license. No changes have been made to the images [139].

Schiavone and Qiu [137] have conducted a comparative study on the mechanical behavior of metallic (Xience) and bioresorbable polymeric (Elixir) stent systems. Their analysis focused on the crimping and deployment processes. The finite element software ABAQUS was used to generate the geometric models and meshes for the balloon, stent, and compromised arterial structure. In ABAQUS, strain hardening was modeled by calculating the yield stress based on the plastic strain, as indicated by the tensile curves. The plaque model was created using the Ogden hyperelastic model, and its behavior was described according to the model parameters. The parameters for the hyperelastic model were determined according to Zahedmanesh and Lally’s research [138]. In this study, the tri-folded balloon was treated as linear elastic material. The material density, Young’s modulus, and Poisson’s ratio were set at 1.1 × 106 kg/mm^3^, 900 MPa, and 0.3, respectively. As illustrated in Figure 6, the biomechanics of the MiStent are demonstrated.

The HGO model computed both longitudinal and circumferential stress–stretch responses, which agreed very well with the experimental data for all three vessel layers. The HGO model will be used in this project to analyze incompressible hyperelastic materials. In order to take compressible deformation into consideration, the HGO-C model will be proposed. The anisotropic part will be expressed by isochoric invariants (which are insensitive to volumetric deformation) [140].

In the study by Schiavone and Qiu [137], the HGO model was employed for the investigation of incompressible hyperelastic materials. The anisotropic part was expressed by isochoric invariants (which are insensitive to volumetric deformation). However, Nolan and collaborators have identified a discrepancy between experimental and simulated compressible anisotropic behavior in the HGO-C model. This inaccuracy is attributed to the employment of isochoric anisotropic invariants, which are unresponsive to volumetric deformation [140]. Consequently, they developed a modified anisotropic (MA) model, utilizing the complete anisotropic invariants to account for volumetric anisotropic contributions. The MA model demonstrated an ability to foresee the material’s anisotropic response to hydrostatic tensile loading, pure shear, and uniaxial deformations with a high degree of accuracy. The HGO-C model was found to substantially underestimate arterial elasticity, a finding with potential ramifications for the simulation outcomes of stent deployment in diseased arteries. To elucidate this effect in its entirety, a substantial volume of new work is necessary, particularly the efforts required for coding a user-defined material subroutine for the MA model (interface with the FE package Abaqus). Nonetheless, the HGO model was utilized and appropriately calibrated against the longitudinal and circumferential tensile data of arteries in conjunction with a CFD model [141]. It has been demonstrated that this model is a reliable anisotropic framework for simulating the deformation of the arterial layers with collagen fiber reinforcement.

**Figure 7:**
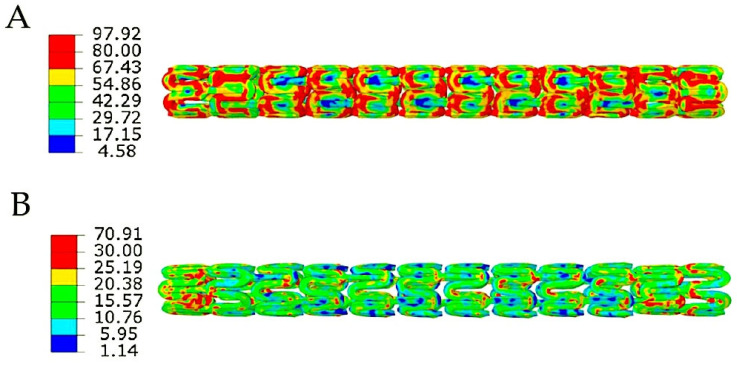
(**A**) The von Mises stress (MPa) contour plot for Elixir (MiStent SES-Elixir Medical Corporation, USA) stent in a fully crimped configuration. (**B**) The MPa contour plot Elixir after springback [120]. This article is distributed under the terms of the Creative Commons Attribution 4.0 International License (http://creativecommons.org/licenses/by/4.0/ 19 July 2026), which permits unrestricted use, distribution, and reproduction in any medium, provided you give appropriate credit to the original author(s) and the source, as well as a link to the Creative Commons license. No changes have been made to the images [139].

According to the study by Schiavone and Qiu [137], a total of 12 rigid plates were generated around the stent in the FEA model to analyze the crimping and deployment phases. These plates were then subjected to forced radial displacement to simulate crimping resulting from stent deployment. The positioning of two stent models was thoroughly evaluated in a rigorous testing procedure. The balloon was inflated to simulate internal pressure, which is an important step in the process. The process allowed for the identification of the rigid contact points between the balloon, the stent, and the diseased artery, providing valuable performance data under realistic conditions. Abaqus Explicit was used for the crimping simulations, with a step time of 0.1 s. The consideration of these constraints was a key factor. It should be noted that there was a degree of hard contact between the stent’s outer surface and the rigid plates. In accordance with the established parameters, the friction coefficient was set to 0.8. After the crimping process is finished, a critical step will be taken to replicate the spring back of the stent within 0.1 s. In this step of the process, the rigid plates are affixed to the stent, thereby enabling the stent to regain its elastic deformation. Figure 7 shows the von Mises stress contour plot for Elixir.

Stress levels were identified as elevated in both polymeric and metallic stents as part of the standard deployment process in arteries [142,143,144,145,146]. Consistent with observations from the crimping process, maximum von Mises stresses were identified in the U-bend regions of both the Xience and the Elixir stents, with values recorded at 935 MPa and 95 MPa, respectively. In arterial contexts, the maximum stresses were typically observed on the plaque, particularly at the edges of the stenosis. It has been determined that this issue is the result of the dogbone-shaped geometry of the implanted stent. Research has shown that stress concentration is linked to localized rupture in plaques [147,148,149]. Studies have identified a higher propensity for plaque rupture to occur at the extremities of the plaque region [142,143]. This condition can lead to significant health concerns, such as heart attacks (coronary arteries) or strokes (carotid arteries) [148,149]. Furthermore, there exists a potential for localized damage to plaques and arteries in high-stress areas. This condition also results in the formation of new tissue, for example, the growth of smooth muscle cells around the stent, which can lead to in-stent restenosis. As illustrated by these findings, the maximum principal stress on the plaque was determined to reach 1.43 MPa and 0.54 MPa for the Xience and Elixir stents, correspondingly [137]. The Elixir stent has been meticulously designed to minimize stress levels, thereby reducing the risk of complications. This objective is accomplished by ensuring optimal compatibility between the polymer and the artery, as well as minimizing arterial expansion.

As indicated by the study of Schiavone et al. [137], there was a clear lack of assessment of stress triaxiality in stents and diseased arteries. Stress triaxiality is defined as the ratio of hydrostatic stress to the equivalent (or von Mises) stress. It is a critical parameter employed in ductile fracture analysis. The study primarily focused on the stress state of the stent–artery system during crimping and expansion, as opposed to fracture or failure. This analysis definitively showed that stress triaxiality is not present. Further research is necessary to investigate this matter in greater depth, with the aim of examining the failure and fracture of stents, plaques, and arteries in the simulations [137].

The expansion procedure was simulated in two stages, which included inflation and deflation, respectively. During the inflation step (0.1 s), a pressure that increases linearly to 1.2 MPa was applied within the balloon. During the deflation step (0.1 s), the balloon pressure was reduced to zero in a linear fashion. All analyses were conducted with consideration for the residual stresses generated during the crimping process. It is important to reiterate that the simulations were executed using the explicit solver in Abaqus. The data outputs offer quantitative measurements of stent expansion, as well as the recoiling and dogboning effects [137,139]. Elixir stent demonstrated a slower expansion profile compared to Xience stent during deployment. Following the deflation of the balloon, the diameter of the stent was found to be smaller for the Elixir stent, due to higher recoiling. The expansion of the Elixir stent resulted in reduced stresses in the plaque and artery. The crimping-generated residual stresses in the simulations resulted in reduced expansion, increased dogboning, and decreased vessel stresses [137,139].

## 5. Discussion

The strategic implementation of ultrathin struts has been identified as a key initiative to enhance PCI outcomes. A number of large-scale randomized trials have been carried out recently to assess the potential benefits of this program. Two substantial systematic meta-analyses have been conducted to examine the disparities between the extant ultrathin platforms. Bangalore et al. [36] conducted a meta-analysis of over 11,500 patients. In the ten-trial study, eight trials assessed the Orsiro SES (5444 patients), one trial assessed the Mistent SES (703 patients), and one trial assessed the Biomime SES (170 patients) [124]. At the one-year follow-up stage, the ultrathin strut DES reduced the risk of TLF by 16% in comparison with a conventional new-generation DES (relative risk, 0.84; 95% CI, 0.72–0.99). The findings proved consistently reliable across trials, with no discernible variations observed according to the type of ultrathin DES employed. The decline in the incidence of TLFs was driven by a decrease in the prevalence of MI, which can be attributed primarily to a decline in ST and peri-operative MI rates [150]. As demonstrated in the subsequent larger meta-analysis, which incorporated 16 randomized trials with 20,701 patients [150,151], these findings were confirmed. The DESs under review included the Orsiro (12 trials, 17,658 patients), the MiStent (2 trials, 1582 patients), the BioMime (1 trial, 256 patients), and the Supraflex (1 trial, 1435 patients). After a follow-up period of 2.5 years, it was found that ultrathin-strut DESs were linked with a reduced risk of TLF and TVF. There was no statistically significant interaction according to stent type in the ultrathin-strut group. As the stent comparison groups in these meta-analyses comprised exclusively newly developed drug-eluting stents (DESs) with biocompatible polymers, the observed differences may be attributable to the reduction of strut thickness by more than 10 mm [152,153]. It is possible that this difference could lead to an enhancement of strut endothelization as a result of a decrease in vessel injury and vascular inflammation, as well as reduced peri-operative myocardial infarction due to less flow disturbance to the side branches [154].

A significant concern in the context of a ST-elevated myocardial infarction (STEMI) is the increased likelihood of early ST, attributable to the prothrombotic environment created by the culprit lesions. The results of the BIOSCIENCE trial demonstrated a lower rate of target lesion failure (TLF) in patients with ST-elevation myocardial infarction (STEMI) who received the Orsiro stent (SES) compared to patients who received the Xience stent (Xience EES). This was evident at both 12- and 24-month follow-ups, with rates of TLF of 3.3% and 5.4% in the Orsiro SES group, as opposed to 8.7% and 10.8% in the Xience EES group, respectively (*p* = 0.024 and *p* = 0.043 at 12 and 24 months, respectively) [155,156,157]. As detailed in the BIOSTEMI report, the Orsiro SES has been shown to be an effective treatment for patients experiencing STEMI. After 12 months, the Orsiro SES was linked with a lower rate of TLF compared to the Xience EES (4% vs. 6%, posterior probability of superiority = 0.986) [71]. The Supraflux Cruz stent has demonstrated positive results in a range of clinical trials, meeting initial outcomes that met expectations in various lesion types and patient demographics [158]. The Supraflex SES has demonstrated its safety and efficacy in two large-scale studies. In the first study, conducted as part of the Talent trial, the Supraflex SES was used in 229 patients suffering from ST-elevation myocardial infarction (STEMI). In the second study, which was based on data from the Flex registry, the Supraflex SES was used in 198 patients. Both studies reported a low incidence of both TLR and stent thrombosis [158,159].

### 5.1. In-Stent Restenosis

ISR, or the narrowing of the stented artery, represents one of the most significant concerns related to stent implantation, as outlined in Ref. [160]. This problem is directly linked to neointima proliferation, which is mainly driven by accumulations of smooth muscle cells and an extracellular matrix [160]. Recent investigative findings have established a strong correlation between the biomechanics of the arterial wall and instances of ISR. The process of stenting has been shown to alter the biomechanical environment, thereby regulating the inflammatory and remodeling processes of vessel walls. It was noted that stent configurations that triggered elevated levels of non-physiological stresses resulted in a more pronounced pathobiological reaction of the vessel walls. This, in turn, led to increased neointimal proliferation [161]. In vitro investigations have demonstrated that mechanical stresses can modulate the growth and movement of vascular cells [162] as well as the production and restructuring of the extracellular matrix [163]. Research has demonstrated a clear correlation between stent-induced vessel stresses and the level of artery injury, with the development of restenosis also promoted. The study clearly demonstrates that the stress levels in the arteries are significantly influenced by the materials and designs of the stents. PLLA, with its reduced modulus, diminished strain hardening, and enhanced strain relaxation, effectively mitigates the interaction between the stent and the artery, leading to a notable reduction in stress levels within the vessel layers. This approach is supported by clinical evidence of its effectiveness. Additionally, bioresorbable polymeric stents exhibit greater flexibility compared to metallic stents, which reduces the risk of vascular responses [164,165].

### 5.2. The Issue of Residual Stress in Stents

The residual stresses present in stents were subjected to rigorous analysis using state-of-the-art X-ray diffraction methodology as outlined in Refs. [139,166]. It was determined that significant microscale stresses could be generated within the device during the crimping process, which has significant implications for the manufacturing process as a whole. As mentioned in the relevant research, the aforementioned phenomenon has also been identified in other studies. Therefore, it is important to consider these findings. The next step was stent expansion, which resulted in increased stress due to tension [137,139]. The level of stress induced by crimping and expansion has the potential to have a significant impact on the fatigue durability of the stent [137,139]. After a thorough review of the available data, it was determined that the crimping and expansion procedures resulted in equivalent levels of stress being observed within the stent struts [139,167,168,169].

In addition, the expansion behavior of the stents during the deployment step was influenced by residual stresses developed during the crimping process, albeit only to a minor extent. The presence of stressed residual material in the device is believed to have enhanced its flexibility. This, in turn, resulted in reduced stress being exerted on the plaque throughout the process of deformation. As outlined in the study by Schiavone and Qiu et al., the hypothesis was validated by the simulation results. However, the impact was found to be relatively negligible [137,139].

### 5.3. Expansion Process: Mechanics and Clinical Setting Analysis

It was determined that the targeted vessel diameter (i.e., 3 mm) could not be achieved through stent expansion alone. This is a relevant consideration in the context of both polymer and metallic stents. The issue was initially attributed to the accelerated expansion of the arterial layers, which emerged during the subsequent phase of vessel stretching. This occurred when the vessel layer stiffness, particularly the intima layer, underwent a significant increase in response to substantial stretch. Secondly, it was assumed that vessel layers would deform purely elastically [137,139]. This assumption resulted in a significant recovery force being exerted on the expanded stent following balloon deflation. In general, the growth of polymer stents has been found to be more gradual than that of metallic stents. The polymeric stent demonstrated higher recoiling, which is attributable to its comparatively less robust mechanical profile. It has come to our attention that there is a significant issue with the use of polymer stents in achieving the desired level of luminal caliber, particularly in cases involving patients with stiffer arteries and advanced calcium plaque formations. It has been demonstrated that polymer stents induce significantly lower stresses in the arteries compared to metallic stents. It is important to note the potential benefits of this approach, which include a reduction in the likelihood of arterial injury and restenosis [133,137,139,142,143].

In clinical settings, the majority of stents undergo post-dilation using balloons that are designed to be fairly non-compliant. These balloons are either stiffer or larger than the balloons used for deployment. This method guarantees optimal adhesion and the precise achievement of the intended diameter [144,145,146,147]. However, it was noted that the simulation did not include the possibility of multiple cycles of inflation. The simulation was conducted as part of a series of studies. In each instance, either a stent was deployed once or the stenting of a vascular lesion was carried out directly. In the medical field, there is a possibility of pre-dilatation of the affected artery. Direct stenting is a widely employed procedural approach. However, its use in combination with bioresorbable polymer stents is comparatively rare [137,139,145,146,147]. In the field of bioresorbable polymer stents and other novel technologies, practitioners generally acknowledge the importance of meticulous lesion pre-dilatation and post-dilatation. The balloon inflation procedure before di-latation modifies the plaque, reducing recoil after the deployment of a stent or scaffold. Following the successful deployment of a stent, the subsequent step involves a post-dilatation procedure. This procedure is typically performed using a second, larger balloon. In some cases, the objective may be to enhance removal efficiency by using a tapered vessel, or to “crack” a particularly resistant plaque [117,139,170,171].

In specific cases, the implementation of two stents with an overlapping segment requires this procedural approach. Achieving optimal results with polymer stents necessitates proper pre-dilatation and post-dilatation procedures. The simulations indicate that larger amounts of recoil and dogboning are to be expected. This suggests that adequate pre-dilatation and post-dilatation are critical for polymeric stents to achieve optimal clinical results [172]. It is essential to note the key differences between inelastic and elastic deformation [173]. A number of studies have centered on simulating pre-dilation—the stretching or bending of a body part—and post-dilation, which is the stretching or bending of a body part after it has been initially stretched or bent. These studies have made the assumption that the artery and the plaque behave elastically, using a hyperelastic model, which is a model that uses a hyper-parameter to represent the material’s elastic behavior. The stresses caused by dilation or post-dilation are eliminated promptly as soon as the dilation pressure is withdrawn. However, to ensure an accurate description of irreversible deformation, both the plaque and the arterial wall require appropriate inelastic or damage models [174,175,176,177,178,179].

Additionally, the maximum tensile stresses in the vessel fibers are usually greater than the ultimate tensile strength of the tissue layer [135]. This text provides an update on the situation regarding the plaques. As Qiu et al. observed [139], the radial stiffness of the bioresorbable Absorb, Elixir, and RevaMedical stents was comparable to that reported by Pauck and Reddy [165]. In their research, the radial stiffness was found to be approximately 500 kPa/mm for a standard polymeric stent (E = 1.8 GPa and yield stress = 45 MPa), and increased to 1300 kPa/mm when the material modulus was increased to 3.6 GPa. These observations suggest the possibility of a link between the material properties of the stents and their radial properties. Additionally, the RevaMedical stent showed superior radial stiffness and strength when compared to the Absorb and Elixir stents, despite the use of a comparable U-bend design. As indicated by this finding, the thickness of the strut also exerts a significant influence on the radial properties of the stent [139].

Therefore, incorporating tissue damage modeling into the finite element simulations at elevated pressure levels is necessary. Tissue impairment has been shown to be associated with irreparable arterial remodeling. This condition can significantly reduce the stent’s recoil upon balloon deflation. It is anticipated that further expansion of the tenting will be necessary in cases where tissue injury is determined to be a result of irreparable mechanical distortion of the arterial plaque system. It is predicted that this would increase the lumen diameter [132,133,142,147,148,180]. Likewise, it is expected that the tenting will extend further in cases where tissue impairment is considered to be a result of irreparable mechanical deformation of the arterial plaque system. This is expected to result in an augmented lumen diameter [177]. Additionally, the use of asymmetric stents has been demonstrated to minimize central radial recoiling and the dogboning phenomenon [180].

### 5.4. Design Evaluation

A thorough design evaluation process is in place to assess various objectives and constraints, including structural, hemodynamic, and other considerations, for different stent designs. This process utilizes state-of-the-art computer simulation techniques, including finite element methods (FEMs) and computational fluid dynamics (CFD), employing specialized software such as ABAQUS and ANSYS (2600 Ansys Drive Canonsburg, PA 15317 USA). These simulations are also used to support sensitivity analysis assessments of performance metrics across various design parameters. FEM is the standard numerical technique used to evaluate structural objectives. The three-dimensional finite element method (3D FEM) is currently considered to be at the forefront of technological advancement in this field [176,181,182,183,184,185]. In contrast, the two-dimensional finite element method (2D FEM) was previously utilized to assess structural objectives in select studies [186,187,188]. In addition to calculating structural metrics, finite element method (FEM) software has been used to evaluate drug transport [184,188], manufacturing processes [185,189,190], and, on one occasion, a hemodynamic objective [191]. In most cases, CFD is the preferred method for hemodynamic objective evaluation. While CFD was used in earlier studies to assess hemodynamic objectives, more advanced three-dimensional CFD assessments are currently preferred. In one evaluation, both the finite element model and the computational fluid dynamics model were utilized for the purpose of assessing drug accumulation and distribution in an artery [184].

To enhance our comprehension of the biomechanical processes associated with devices implanted in various locations, such as coronary vessels, aorta, pulmonary arteries, and ventricular structures, when assessed using FEM models, further investigation is necessary. Further investigation is recommended to develop models of arterial layers for the study of biomechanical features and varying pressure conditions [192,193], along with right and left ventricular structures, such as papillary muscles [194,195].

With respect to the stents implanted in coronary sites, the composition of the plaque dictates its classification. Plaques are typically classified as hypocellular, cellular, or calcified. The management of calcified plaques represents a significant challenge. This is due to the inherent resistance of calcified plaques to stretching, in comparison to hypocellular or cellular plaques [142]. To date, the impact of plaque structure on stent expansion has been assessed solely in the context of an isotropic tissue model. Further research is required to investigate these effects in the context of vessel anisotropy. Finally, it is important to note that polymers generally exhibit isotropy and visco-plasticity. These properties may also have an impact on the results of the simulation. This issue must be addressed in subsequent investigations [179].

## 6. Conclusions

The advent of ultrathin struts DESs signaled a major advancement in PCI technology, redirecting its emphasis toward bolstering patient safety and efficacy. Extensive clinical trials have shown that ultrathin struts stents are the optimal stent choice in numerous situations and for various types of lesions. Ongoing randomized trials will provide more robust and reliable data on the effectiveness and reliability of ultrathin DES. Employing finite element analysis (FEA) allows for rapid comprehension of the crimping and deployment of polymeric and metallic stents. This enables us to make direct comparisons between their mechanical behaviors. The findings showed that the polymer stent expanded at a reduced rate compared to the metallic stent. Overall expansion of the polymer stent was lower when subjected to both peak inflation and subsequent deflation due to the weaker material properties exhibited by the material. This phenomenon is also associated with the polymer device’s higher recoiling effect. It is important to note that utilizing polymer stents in treating patients with heavily calcified plaques or stiff vessels may require pre-dilation or post-dilation, which can pose a challenge in certain cases. During the procedure, the application of force through crimping can generate significant levels of residual stresses in the stent. These stresses can impact the stent’s expansion and result in increased ogboning for Elixir polymer devices. Nevertheless, no modifications were made to the stress redistribution process during the deployment phase, with only minor adjustments being implemented to stress magnitude.

## Figures and Tables

**Table 1:** Narrative review searching strategies [31].

Items	Specification
Date of Search (specified to date, month and year)	From July 2025 to January 2026
Databases and other sources searched	PubMed, MEDLINE, Embase, and the Cochrane Library
Search terms used (including MeSH and free text search terms and filters)	Drug-eluting stents; percutaneous coronary intervention; stent restenosis; ultrathin-strut DESs; computational modeling and finite element analysis; combined with “STEMI,” “NSTEMI,” “crimping,” “deployment,” “recoil,” and “dogboning.”
Timeframe	Up to July 2025
Inclusion and exclusion criteria (study type, language restrictions, etc.)	English language; inclusion criteria: the manucripts under consideration here all concern cases of PCI using DESs (ultrathin struts and not) with and without BP
Selection process	Two authors independently selected articles after screening for duplicates

**Table 2:** Drug-eluting stents (DESs) with and without Biopolimer.

	Orsirio (Biotronic)	SYNERGY (Boston Scientific)	Auxetic NITFE (US Patent No. 10,765,778 B2; European Patent European Grant E5838@E0109459	XIENCE (Abbot)
Platform size (mm)	2.5 to 4.0	2.25 to 5.0	2.20 to 5.25	2.25 to 5.25
Strut thickness (µm)	Co-Cr L605 60 to 80	Pt-Cr 74 to 81	Auxetic Ni-Ti + expanded PTFE Auxetic ferromagnetic + expanded PTFE	Co-Cr L605 + fluoro polymer **
Crossing profile (mm)	0.95		Auxetic 0.80 to 1.0	
Avaiable diameters (mm)	2.25 to 4.0	2.25 to 5.0	2.20 to 5.25	2.22 to 5.25
Avaible lenghts (mm)	9 to 40	8 to 38	8 to 50	12 to 48
Polymer type	proBIO * - PLLA sirolimus	Biodegradable - PLGA Everolimus	Auxetic BIO * - PLLA Sirolimus - PLGA Sirolimus - PHAs Sirolimus	Fluorinated surface against CD42b/CD61
Polymer thickness (µm)	3.5 abluminal 7.5 luminal	4.0 abluminal	4.0 abluminal 15 luminal	4.0 luminal
Polymer absorption	15 mths	4 mths	4 mths 24 mths	No absorption
Eluted drug	Sirolimus	Everolimus	Sirolimus	Everolimus
Drug dose	1.4 µg/mm^2^	1.0 µg/mm^2^	1.4 µg/mm^2^ to 2.4 µg/mm^2^ (microcrystals).	1.0 µg/mm^2^
Drug release	50% by 1 mth 80% by 3 mths	3 mths (at most 4 mths)	50% by 1 mths 80% by 9 mths	1 mth to 4 mths

Abbreviations: Cobalt–Chromium L605, Co-Cr L605; Nickel–Titanium, Ni-Ti; month, mth; Platinum–Chromium, Pt-Cr; PLGA, poly-D, L-lactide-co-glycolide; PLLA, poly-L-lactic acid; PHAs, polyhydroxyalkanoates; * Biodegradable; ** Thromboresistance and low inflammatory responses [38,40,44,45,54,55,56,57,58,63,64,65,66,67].

**Table 3:** Features of the Auxetic UtS-DES.

Auxetic UtS-DES (US Patent No. 10,765,778 B2; European Patent European Grant E5838@E0109459).										
Platform Size (mm)	Strut Thickness (µm)	Crossing Profile	Avaiable Diameters (mm)	Avaible Lenghts (mm)	Polymer Type	Polymer Material and Thickness	Polymer Absorption	Eluted Drug	Drug Dose	Drug Release
2.20 mm to 4.25 mm 2.20 mm to 5.25 mm	Auxtic titanium–nickel alloy plus expanded PTFE Auxetic ferromagnetic alloy plus expanded PTFE	Auxetic 0.80 mm to 1.0 mm	2.20; 2.5; 2.75; 3.0; 3.5; 4.0; 4.5; 5.25	8 mm to 50 mm	Biodegradable (auxetic BIO) - PLLA Sirolimus - PLGA Sirolimus - PHAs Sirolimus	4 µm abluminal 15 µm luminal	4 months 24 months	Sirolimus	1.4 µg/mm^2^ to 2.4 µg/mm^2^ (microcrystals)	50% 1 month 80% 9 months

**Table 4:** Parameter values of the anisotropic Holzapfel–Gasser–Ogden model for the arterial layers.

Layer	p (kgmm^3^)	*C*_10_	*D*	*k*_1_	*k*_2_	k	g
Intima	1.066 × 10^−6^	0.03	8.95 × 10^−7^	4	1200.0	0.303	60°
Media	1.066 × 10^−6^	0.005	5.31 × 10^−6^	0.57	80.0	0.313	20°
Adventitia	1.066 × 10^−6^	8.32 × 10^−3^	4.67 × 10^−6^	1.0	1000.0	0.303	67°

## Data Availability

Dataset available on request from the authors.

## References

[1] Nappi F., Nenna A., Chello M. (2022). Structural Heart Valve Disease in the Era of Change and Innovation: The Crosstalk between Medical Sciences and Engineering. Bioengineering.

[2] Nappi F., Avtaar Singh S.S., Nappi P., Fiore A. (2022). Biomechanics of Transcatheter Aortic Valve Implant. Bioengineering.

[3] Nappi F., Attias D., Avtaar Singh S.S., Prot V. (2019). Finite element analysis applied to the transcatheter mitral valve therapy: Studying the present, imagining the future. J. Thorac. Cardiovasc. Surg..

[4] Piccolo R., Giustino G., Mehran R., Windecker S. (2015). Stable coronary artery disease: Revascularisation and invasive strategies. Lancet.

[5] Beier S., Ormiston J., Webster M., Cater J., Norris S., Medrano-Gracia P., Young A., Cowan B. (2016). Hemodynamics in Idealized Stented Coronary Arteries: Important Stent Design Considerations. Ann. Biomed. Eng..

[6] Gharleghi R., Zhang M., Shen C., Webster M., Ellis C., Beier S. (2025). Assessing left main bifurcation anatomy and haemodynamics as a potential surrogate for disease risk in suspected coronary artery disease without stenosis. Sci. Rep..

[7] Medrano-Gracia P., Ormiston J., Webster M., Beier S., Ellis C., Wang C., Young A.A., Cowan B.R. (2014). Construction of a Coronary Artery Atlas from CT Angiography. Medical Image Computing and Computer-Assisted Intervention–MICCAI 2014.

[8] Medrano-Gracia P., Ormiston J., Webster M., Beier S., Ellis C., Wang C., Smedby Ö., Young A., Cowan B. (2017). A Study of Coronary Bifurcation Shape in a Normal Population. J. Cardiovasc. Transl. Res..

[9] Craiem D., Casciaro M.E., Graf S., Glaser C.E., Gurfinkel E.P., Armentano R.L. (2009). Coronary arteries simplified with 3D cylinders to assess true bifurcation angles in atherosclerotic patients. Cardiovasc. Eng..

[10] Metz C.T., Schaap M., Klein S., Neefjes L.A., Capuano E., Schultz C., van Geuns R.J., Serruys P.W., van Walsum T., Niessen W.J. (2009). Patient specific 4D coronary models from ECG-gated CTA data for intra-operative dynamic alignment of CTA with X-ray images. Medical Image Computing and Computer-Assisted Intervention—MICCAI 2009.

[11] Points Forts de la Nouvelle Version R2026a—MATLAB et Simulink. https://fr.mathworks.com/products/new_products/latest_features.html.

[12] Hildick-Smith D., Arunothayaraj S., Stankovic G., Chen S.L. (2022). Percutaneous coronary intervention of bifurcation lesions. EuroIntervention.

[13] Sheiban I., Figini F., Gasparetto V., D’Ascenzo F., Moretti C., Leonardo F. (2021). Side Branch is the Main Determinant Factor of Bifurcation Lesion Complexity: Critical Review with a Proposal Based on Single-centre Experience. Heart Int..

[14] Murasato Y., Meno K., Mori T., Tanenaka K. (2022). Impact of coronary bifurcation angle on the pathogenesis of atherosclerosis and clinical outcome of coronary bifurcation intervention-A scoping review. PLoS ONE.

[15] Dzavik V., Kharbanda R., Ivanov J., Ing D.J., Bui S., Mackie K., Ramsamujh R., Barolet A., Schwartz L., Seidelin P.H. (2006). Predictors of long-term outcome after crush stenting of coronary bifurcation lesions: Importance of the bifurcation angle. Am. Heart J..

[16] Ungureanu C., Natalis A., Cocoi M., Dumitrascu S., Noterdaeme T., Gach O., Jossart A., Soetens R., Colletti G. (2024). The impact of the bifurcation angle for the Nano-Crush two-stent coronary bifurcation technique on long-term outcomes in a real-world clinical population. Cardiovasc. Revascularization Med..

[17] Girasis C., Schuurbiers J.C., Muramatsu T., Aben J.P., Onuma Y., Soekhradj S., Morel M.A., van Geuns R.J., Wentzel J.J., Serruys P.W. (2013). Advanced three-dimensional quantitative coronary angiographic assessment of bifurcation lesions: Methodology and phantom validation. EuroIntervention.

[18] Grundeken M.J., Ishibashi Y., Ramcharitar S., Tuinenburg J.C., Reiber J.H., Tu S., Aben J.P., Girasis C., Wykrzykowska J.J., Onuma Y. (2015). The need for dedicated bifurcation quantitative coronary angiography (QCA) software algorithms to evaluate bifurcation lesions. EuroIntervention.

[19] Zahnd G., Schrauwen J., Karanasos A., Regar E., Niessen W., van Walsum T., Gijsen F. (2016). Fusion of fibrous cap thickness and wall shear stress to assess plaque vulnerability in coronary arteries: A pilot study. Int. J. Comput. Assist. Radiol. Surg..

[20] Tomaniak M., Masdjedi K., Neleman T., Kucuk I.T., Vermaire A., van Zandvoort L.J.C., Van Boven N., van Dalen B.M., Soei L.K., den Dekker W.K. (2022). Three-dimensional QCA-based vessel fractional flow reserve (vFFR) in Heart Team decision-making: A multicentre, retrospective, cohort study. BMJ Open.

[21] Suzuki N., Asano T., Nakazawa G., Aoki J., Tanabe K., Hibi K., Ikari Y., Kozuma K. (2020). Clinical expert consensus document on quantitative coronary angiography from the Japanese Association of Cardiovascular Intervention and Therapeutics. Cardiovasc. Interv. Ther..

[22] Piccolo R., Pilgrim T., Heg D., Franzone A., Rat-Wirtzler J., Räber L., Silber S., Serruys P.W., Jüni P., Windecker S. (2015). Comparative Effectiveness and Safety of New-Generation Versus Early-Generation Drug-Eluting Stents According to Complexity of Coronary Artery Disease: A Patient-Level Pooled Analysis of 6081 Patients. JACC Cardiovasc. Interv..

[23] Piccolo R., Franzone A., Windecker S. (2018). From bare metal to barely anything: An update on coronary stenting. Heart.

[24] Hoffmann R., Mintz G.S. (2000). Coronary in-stent restenosis—Predictors, treatment and prevention. Eur. Heart J..

[25] Tada T., Byrne R.A., Simunovic I., King L.A., Cassese S., Joner M., Fusaro M., Schneider S., Schulz S., Ibrahim T. (2013). Risk of Stent Thrombosis Among Bare-Metal Stents, First-Generation Drug-Eluting Stents, and Second-Generation Drug-Eluting Stents. JACC Cardiovasc. Interv..

[26] Vranckx P., Kint P.P., Morel M.A., Van Es G.A., Serruys P.W., Cutlip D.E. (2008). Identifying stent thrombosis, a critical appraisal of the academic research consortium (ARC) consensus definitions: A lighthouse and as a toe in the water. EuroIntervention.

[27] Piccolo R., Bonaa K.H., Efthimiou O., Varenne O., Baldo A., Urban P., Kaiser C., Remkes W., Räber L., de Belder A. (2019). Coronary Stent Trialists’ Collaboration. Drug-Eluting or Bare-Metal Stents for Percutaneous Coronary Intervention: A Systematic Review and Individual Patient Data Meta-Analysis of Randomised Clinical Trials. Lancet.

[28] Piscione F., Piccolo R., Cassese S., Galasso G., Chiariello M. (2009). Clinical Impact of Sirolimus-Eluting Stent in ST-Segment Elevation Myocardial Infarction: A Meta-Analysis of Randomized Clinical Trials. Catheter. Cardiovasc. Interv..

[29] Neumann F.-J., Sousa-Uva M., Ahlsson A., Alfonso F., Banning A.P., Benedetto U., Byrne R.A., Collet J.-P., Falk V., Head S.J. (2019). 2018 ESC/EACTS Guidelines on Myocardial Revascularization. Eur. Heart J..

[30] Kim W., Jeong M.H., Cha K.S., Hyun D.W., Hur S.H., Kim K.B., Hong Y.J., Park H.W., Kim J.H., Ahn Y.K. (2005). Effect of Anti-Oxidant (Carvedilol and Probucol) Loaded Stents in a Porcine Coronary Restenosis Model. Circ. J..

[31] Baethge C., Goldbeck-Wood SandMertens S. (2019). SANRA-a scale for the quality assessment of narrative review articles. Res. Integr. Peer Rev..

[32] Strauss B.H., Tanguay J.F., Picard F., Doucet S., Morice M.C., Elbaz-Greener G. (2022). Coronary Stenting: Reflections on a 35-Year Journey. Can. J. Cardiol. Can. J. Cardiol..

[33] Iqbal J., Gunn J., Serruys P.W. (2013). Coronary stents: Historical development, current status and future direction. Br. Med. Bull..

[34] Sigwart U., Puel J., Mirkovitch V., Joffre F., Kappenberger L. (1987). Intravascular Stents to Prevent Occlusion and Re-Stenosis after Transluminal Angioplasty. N. Engl. J. Med..

[35] Serruys P.W., de Jaegere P., Kiemeneij F., Macaya C., Rutsch W., Heyndrickx G., Emanuelsson H., Marco J., Legrand V., Materne P. (1994). A Comparison of Balloon-Expandable-Stent Implantation with Balloon Angioplasty in Patients with Coronary Artery Disease. N. Engl. J. Med..

[36] Moses J.W., Leon M.B., Popma J.J., Fitzgerald P.J., Holmes D.R., O’Shaughnessy C., Caputo R.P., Kereiakes D.J., Williams D.O., Teirstein P.S. (2003). Sirolimus-eluting stents versus standard stents in patients with stenosis in a native coronary artery. N. Engl. J. Med..

[37] Camenzind E., Steg P.G., Wijns W. (2007). Stent thrombosis late after implantation of first-generation drug-eluting stents: A cause for concern. Circulation.

[38] Bangalore S., Toklu B., Patel N., Feit F., Stone G.W. (2018). Newer-Generation Ultrathin Strut Drug-Eluting Stents Versus Older Second-Generation Thicker Strut Drug-Eluting Stents for Coronary Artery Disease. Circulation.

[39] Navarese E.P., Kowalewski M., Kandzari D., Lansky A., Górny B., Kołtowski L., Waksman R., Berti S., Musumeci G., Limbruno U. (2014). First-generation versus second-generation drug-eluting stents in current clinical practice: Updated evidence from a comprehensive meta-analysis of randomised clinical trials comprising 31 379 patients. Open Heart.

[40] Kandzari D.E., Mauri L., Koolen J.J., Massaro J.M., Doros G., Garcia-Garcia H.M., Bennett J., Roguin A., Gharib E.G., Cutlip D.E. (2017). Ultrathin, bioresorbable polymer sirolimus-eluting stents versus thin, durable polymer everolimus-eluting stents in patients undergoing coronary revascularisation (BIOFLOW V): A randomised trial. Lancet.

[41] Räber L., Magro M., Stefanini G.G., Kalesan B., van Domburg R.T., Onuma Y., Wenaweser P., Daemen J., Meier B., Jüni P. (2012). Very Late Coronary Stent Thrombosis of a Newer-Generation Everolimus-Eluting Stent Compared with Early-Generation Drug-Eluting Stents. Circulation.

[42] McCreanor V., Parsonage W.A., Whiteman D.C., Olsen C., Barnett A.G., Graves N. (2019). Pharmaceutical use and costs in patients with coronary artery disease, using Australian observational data. BMJ Open.

[43] Kinlay S., Young M.M., Sherrod R., Gagnon D.R. (2023). Long-Term Outcomes and Duration of Dual Antiplatelet Therapy After Coronary Intervention With Second-Generation Drug-Eluting Stents: The Veterans Affairs Extended DAPT Study. J. Am. Heart Assoc..

[44] Iglesias J.F., Roffi M., Degrauwe S., Secco G.G., Aminian A., Windecker S., Pilgrim T. (2017). Orsiro cobalt-chromium sirolimus-eluting stent: Present and future perspective. Expert Rev. Med. Devices.

[45] Blum M., Cao D., Mehran R. (2020). Device profile of the Resolute Onyx Zotarolimus eluting coronary stent system for the treatment of coronary artery disease: Overview of its safety and efficacy. Expert Rev. Med. Devices.

[46] Stone G.W., Ellis S.G., Cox D.A., Hermiller J., O’Shaughnessy C., Mann J.T., Turco M., Caputo R., Bergin P., Greenberg J. (2004). A polymer-based, paclitaxel-eluting stent in patients with coronary artery disease. N. Engl. J. Med..

[47] Schömig A., Dibra A., Windecker S., Mehilli J., Suárez de Lezo J., Kaiser C., Park S.J., Goy J.J., Lee J.H., Di Lorenzo E. (2007). A Meta-Analysis of 16 Randomized Trials of Sirolimus-Eluting Stents Versus Paclitaxel-Eluting Stents in Patients With Coronary Artery Disease. J. Am. Coll. Cardiol..

[48] Torii S., Jinnouchi H., Sakamoto A., Kutyna M., Cornelissen A., Kuntz S., Guo L., Mori H., Harari E., Paek K.H. (2020). Drug-eluting coronary stents: Insights from preclinical and pathology studies. Nat. Rev. Cardiol..

[49] Ahadi F., Azadi M., Biglari M., Bodaghi M., Khaleghian A. (2023). Evaluation of coronary stents: A review of types, materials, processing techniques, design, and problems. Heliyon.

[50] Vishnu J., Manivasagam G., Mantovani D., Udduttula A., Coathup M.J., Popat K.C., Ren P.G., Prashanth K.G. (2022). Balloon expandable coronary stent materials: A systematic review focused on clinical success. In Vitro Models.

[51] Jiang W., Zhao W., Zhou T., Wang L., Qiu T. (2022). A Review on Manufacturing and Post-Processing Technology of Vascular Stents. Micromachines.

[52] Hassan A.K.M., Bergheanu S.C., Stijnen T., van der Hoeven B.L., Snoep J.D., Plevier J.W., Schalij M.J., Wouter Jukema J. (2010). Late stent malapposition risk is higher after drug-eluting stent compared with bare-metal stent implantation and associates with late stent thrombosis. Eur. Heart J..

[53] Tenekecioglu E., Farooq V., Bourantas C.V., Silva R.C., Onuma Y., Yılmaz M., Serruys P.W. (2016). Bioresorbable scaffolds: A new paradigm in percutaneous coronary intervention. BMC Cardiovasc. Disord..

[54] Ang H.Y., Bulluck H., Wong P., Venkatraman S.S., Huang Y., Foin N. (2017). Bioresorbable stents: Current and upcoming bioresorbable technologies. Int. J. Cardiol..

[55] Kereiakes D.J., Ellis S.G., Metzger D.C., Caputo R.P., Rizik D.G., Teirstein P.S., Litt M.R., Kini A., Kabour A., Marx S.O. (2019). Clinical Outcomes Before and After Complete Everolimus- Eluting Bioresorbable Scaffold Resorption. Circulation.

[56] Jinnouchi H., Torii S., Sakamoto A., Kolodgie F.D., Virmani R., Finn A.V. (2019). Fully bioresorbable vascular scaffolds: Lessons learned and future directions. Nat. Rev. Cardiol..

[57] Peng X., Qu W., Jia Y., Wang Y., Yu B., Tian J. (2020). Bioresorbable Scaffolds: Contemporary Status and Future Directions. Front. Cardiovasc. Med..

[58] Kawashima H., Ono M., Kogame N., Takahashi K., Chang C.C., Hara H., Gao C., Wang R., Tomaniak M., Modolo R. (2020). Drug-eluting bioresorbable scaffolds in cardiovascular disease, peripheral artery and gastrointestinal fields: A clinical update. Expert Opin. Drug Deliv..

[59] Cerrato E., Barbero U., Gil Romero J.A., Quadri G., Mejia-Renteria H., Tomassini F., Ferrari F., Varbella F., Gonzalo N., Escaned J. (2019). Magmaris™ _resorbable magnesium scaffold: State-of-art review. Future Cardiol..

[60] Maleckis K., Anttila E., Aylward P., Poulson W., Desyatova A., MacTaggart J., Kamenskiy A. (2018). Nitinol Stents in the Femoropopliteal Artery: A Mechanical Perspective on Material, Design, and Performance. Ann. Biomed. Eng..

[61] Corrales M., Doizi S., Barghouthy Y., Kamkoum H., Somani B., Traxer O. (2021). A systematic review of long-duration stents for ureteral stricture: Which one to choose?. World J. Urol..

[62] Tian S., Huang H., Hu Z., Dong Y., Bai C. (2022). A narrative review of progress in airway stents. J. Thorac. Dis..

[63] Kamberi M., Pinson D., Pacetti S., Perkins L.E.L., Hossainy S., Mori H., Rapoza R.J., Kolodgie F., Virmani R. (2018). Evaluation of chemical stability of polymers of XIENCE everolimus-eluting coronary stents in vivo by pyrolysis-gas chromatography/mass spectrometry. J. Biomed. Mater. Res. Part B Appl. Biomater..

[64] Zanchin C., Ueki Y., Zanchin T., Häner J., Otsuka T., Stortecky S., Koskinas K.C., Siontis G.C.M., Praz F., Moschovitis A. (2019). Everolimus-Eluting Biodegradable Polymer Versus Everolimus-Eluting Durable Polymer Stent for Coronary Revascularization in Routine Clinical Practice. JACC Cardiovasc. Interv..

[65] Serruys P.W., Silber S., Garg S., van Geuns R.J., Richardt G., Buszman P.E., Kelbaek H., van Boven A.J., Hofma S.H., Linke A. (2010). Comparison of zotarolimus-eluting and everolimus-eluting coronary stents. N. Engl. J. Med..

[66] Shiomi H., Kozuma K., Morimoto T., Kadota K., Tanabe K., Morino Y., Akasaka T., Abe M., Takeji Y., Suwa S. (2019). 7-Year Outcomes of a Randomized Trial Comparing the First-Generation Sirolimus-Eluting Stent Versus the New-Generation Everolimus-Eluting Stent: The RESET Trial. RESET Investigators. JACC Cardiovasc. Interv..

[67] Kufner S., Joner M., Thannheimer A., Hoppmann P., Ibrahim T., Mayer K., Cassese S., Laugwitz K.L., Schunkert H., Kastrati A. (2019). Ten-Year Clinical Outcomes From a Trial of Three Limus-Eluting Stents with Different Polymer Coatings in Patients with Coronary Artery Disease. Circulation.

[68] Massberg S., Byrne R.A., Kastrati A., Schulz S., Pache J., Hausleiter J., Ibrahim T., Fusaro M., Ott I., Schömig A. (2011). Polymer-free sirolimus- and probucol-eluting versus new generation zotarolimus-eluting stents in coronary artery disease: The Intracoronary Stenting and Angiographic Results: Test Efficacy of Sirolimus-and Probucol-Eluting versus Zotarolimus-eluting Stents (ISAR-TEST 5) trial. Circulation.

[69] Kufner S., Sorges J., Mehilli J., Cassese S., Repp J., Wiebe J., Lohaus R., Lahmann A., Rheude T., Ibrahim T. (2016). Randomized Trial of Polymer-Free Sirolimus- and Probucol-Eluting Stents Versus Durable Polymer Zotarolimus-Eluting Stents: 5-Year Results of the ISAR-TEST-5 Trial. JACC Cardiovasc. Interv..

[70] Kufner S., Ernst M., Cassese S., Joner M., Mayer K., Colleran R., Koppara T., Xhepa E., Koch T., Wiebe J. (2020). 10-Year Outcomes From a Randomized Trial of Polymer-Free Versus Durable Polymer Drug-Eluting Coronary Stents. J. Am. Coll. Cardiol..

[71] Iglesias J.F., Muller O., Zuffi A., Eeckhout E. (2016). Performance of the Orsiro Hybrid Drug-Eluting Stent in High-Risk Subgroups. Minerva. Cardioangiol..

[72] Gao C., Kogame N., Sharif F., Smits P.C., Tonino P., Hofma S., Moreno R., Choudhury A., Petrov I., Cequier A. (2021). Prospective Multicenter Randomized All-Comers Trial to Assess the Safety and Effectiveness of the Ultra-Thin Strut Sirolimus-Eluting Coronary Stent Supraflex: Two-Year Outcomes of the TALENT Trial. Circ. Cardiovasc. Interv..

[73] Winter R.d.W.d., Zaman A., Hara H., Gao C., Ono M., Garg S., Smits P.C., Tonino P.A.L., Hofma S.H., Moreno R. (2022). Sirolimus-Eluting Stents with Ultrathin Struts versus Everolimus-Eluting Stents for Patients Undergoing Percutaneous Coronary Intervention: Final Three-Year Results of the TALENT Trial. EuroIntervention.

[74] Windecker S., Haude M., Neumann F.-J., Stangl K., Witzenbichler B., Slagboom T., Sabaté M., Goicolea J., Barragan P., Cook S. (2015). Comparison of a Novel Biodegradable Polymer Sirolimus-Eluting Stent with a Durable Polymer Everolimus-Eluting Stent: Results of the Randomized BIOFLOW-II Trial. Circ. Cardiovasc. Interv..

[75] Wijns W., Vrolix M., Verheye S., Schoors D., Slagboom T., Gosselink M., Benit E., Kandzari D., Donohoe D., Ormiston J.A. (2018). Long-Term Clinical Outcomes of a Crystalline Sirolimus-Eluting Coronary Stent with a Fully Bioabsorbable Polymer Coating: Five-Year Outcomes from the DESSOLVE I and II Trials. EuroIntervention.

[76] Dani S., Costa R.A., Joshi H., Shah J., Pandya R., Virmani R., Sheiban I., Bhatt S., Abizaid A. (2013). First-in-Human Evaluation of the Novel BioMime Sirolimus-Eluting Coronary Stent with Bioabsorbable Polymer for the Treatment of Single de Novo Lesions Located in Native Coronary Vessels-Results from the MeriT-1 Trial. EuroIntervention.

[77] Jain R.K., Chakravarthi P., Shetty R., Ramchandra P., Polavarapu R.S., Wander G.S., Mohan B., Banker D.N., Dharmadhikari A., Bansal S.S. (2016). One-Year Outcomes of a BioMimeTM Sirolimus-Eluting Coronary Stent System with a Biodegradable Polymer in All-Comers Coronary Artery Disease Patients: The MeriT-3 Study. Indian Heart J..

[78] Abizaid A., Kedev S., Kedhi E., Talwar S., Erglis A., Hlinomaz O., Masotti M., Fath-Ordoubadi F., Lemos P.A., Milewski K. (2018). Randomised Comparison of a Biodegradable Polymer Ultra-Thin Sirolimus-Eluting Stent versus a Durable Polymer Everolimus-Eluting Stent in Patients with de Novo Native Coronary Artery Lesions: The MeriT-V Trial. EuroIntervention.

[79] Nappi F., Spadaccio C., Castaldo C., Di Meglio F., Nurzynska D., Montagnani S., Chello M., Acar C. (2014). Reinforcement of the pulmonary artery autograft with a polyglactin and polydioxanone mesh in the Ross operation: Experimental study in growing lamb. J. Heart Valve Dis..

[80] Nappi F., Spadaccio C., Fouret P., Hammoudi N., Chachques J.C., Chello M., Acar C. (2015). An experimental model of the Ross operation: Development of resorbable reinforcements for pulmonary autografts. J. Thorac. Cardiovasc. Surg..

[81] Nappi F., Spadaccio C., Fraldi M., Montagnani S., Fouret P., Chachques J.C., Acar C. (2016). A composite semiresorbable armoured scaffold stabilizes pulmonary autograft after the Ross operation: Mr Ross’s dream fulfilled. J. Thorac. Cardiovasc. Surg..

[82] Spadaccio C., Montagnani S., Acar C., Nappi F. (2015). Introducing bioresorbable scaffolds into the show. A potential adjunct to resuscitate Ross procedure. Int. J. Cardiol..

[83] Nappi F., Carotenuto A.R., Di Vito D., Spadaccio C., Acar C., Fraldi M. (2016). Stress-shielding, growth and remodeling of pulmonary artery reinforced with copolymer scaffold and transposed into aortic position. Biomech. Model. Mechanobiol..

[84] Nappi F., Carotenuto A.R., Cutolo A., Fouret P., Acar C., Chachques J.C., Fraldi M. (2016). Compliance mismatch and compressive wall stresses drive anomalous remodelling of pulmonary trunks reinforced with Dacron grafts. J. Mech. Behav. Biomed. Mater..

[85] Nappi F., Fraldi M., Spadaccio C., Carotenuto A.R., Montagnani S., Castaldo C., Chachques J.C., Acar C. (2016). Biomechanics drive histological wall remodeling of neoaortic root: A mathematical model to study the expression levels of ki 67, metalloprotease, and apoptosis transition. J. Biomed. Mater. Res. Part A.

[86] Nappi F., Avtaar Singh S.S. (2022). Biomechanics of Pulmonary Autograft as Living Tissue: A Systematic Review. Bioengineering.

[87] Nappi F., Avtaar Singh S.S., Acar C. (2020). Biomechanical future of the growing pulmonary autograft in Ross operation. Transl. Pediatr..

[88] Utsunomia C., Ren Q., Zinn M. (2020). Poly(4-hydroxybutyrate): Current state and perspectives. Front. Bioeng. Biotechnol..

[89] Doi Y., Segawa A., Kunioka M. (1990). Biosynthesis and characterization of poly(3-hydroxybutyrate-co-4-hydroxybutyrate) in Alcaligenes eutrophus. Int. J. Biol. Macromol..

[90] Huong K.H., Teh C.H., Amirul A.A. (2017). Microbial-based synthesis of highly elastomeric biodegradable poly(3-hydroxybutyrate-co-4-hydroxybutyrate) thermoplastic. Int. J. Biol. Macromol..

[91] Murayama A., Yoneda H., Maehara A., Shiomi N., Hirata H. (2023). A highly elastic absorbable monofilament suture fabricated from poly(3-hydroxybutyrate-co-4-hydroxybutyrate). Sci. Rep..

[92] Girasis C., Farooq V., Diletti R., Muramatsu T., Bourantas C.V., Onuma Y., Holmes D.R., Feldman T.E., Morel M.A., van Es G.A. (2013). Impact of 3-dimensional bifurcation angle on 5-year outcome of patients after percutaneous coronary intervention for left main coronary artery disease: A substudy of the SYNTAX trial (synergy between percutaneous coronary intervention with taxus and cardiac surgery). JACC Cardiovasc. Interv..

[93] Godino C., Al-Lamee R., Rosa C.L., Morici N., Latib A., Ielasi A., Mario C.D., Sangiorgi G.M., Colombo A. (2010). Coronary left main and non-left main bifurcation angles: How are the angles modibed by different bifurcation stenting techniques?. J. Interv. Cardiol..

[94] Rubinshtein R., Lerman A., Spoon D.B., Rihal C.S. (2012). Anatomic features of the left main coronary artery and factors associated with its bifurcation angle: A 3-dimensional quantitative coronary angiographic study. Catheter. Cardiovasc. Interv..

[95] Kawasaki T., Koga H., Serikawa T., Orita Y., Ikeda S., Mito T., Gotou Y., Shintani Y., Tanaka A., Tanaka H. (2009). The bifurcation study using 64 multislice computed tomography. Catheter. Cardiovasc. Interv..

[96] Peederer T., Ludwig J., Ropers D., Daniel W.G., Achenbach S. (2006). Measurement of coronary artery bifurcation angles by multidetector computed tomography. Investig. Radiol..

[97] Medrano-Gracia P., Ormiston J., Webster M., Beier S., Young A., Ellis C., Wang C., Smedby Ö., Cowan B. (2016). A computational atlas of normal coronary artery anatomy. EuroIntervention.

[98] Nappi F., Mazzocchi L., Spadaccio C., Attias D., Timofeva I., Macron L., Iervolino A., Morganti S., Auricchio F. (2021). CoreValve vs. Sapien 3 Transcatheter Aortic Valve Replacement: A Finite Element Analysis Study. Bioengineering.

[99] Yushkevich P.A., Piven J., Hazlett H.C., Smith R.G., Ho S., Gee J.C., Gerig G. (2006). User-guided 3D active contour segmentation of anatomical structures: Significantly improved efficiency and reliability. Neuroimage.

[100] Masdjedi K., Tanaka N., Van Belle E., Porouchani S., Linke A., Woitek F.W., Bartorelli A.B., Ali Z.A., den Dekker W.K., Wilschut J. (2022). Vessel fractional flow reserve (vFFR) for the assessment of stenosis severity: The FAST II study. EuroIntervention.

[101] Gnyaneshwar R., Kumar R.K., Balakrishnan K.R. (2002). Dynamic analysis of the aortic valve using a finite element model. Ann. Thorac. Surg..

[102] Capelli C., Bosi G.M., Cerri E., Nordmeyer J., Odenwald T., Bonhoeffer P., Migliavacca F., Taylor A.M., Schievano S. (2012). Patient-specific simulations of transcatheter aortic valve stent implantation. Med. Biol. Eng. Comput..

[103] Selvadurai A.P.S. (2006). Deflections of a rubber membrane. J. Mech. Phys. Solids.

[104] Yeoh O.H. (1993). Some forms of the strain energy function for rubber. Rubber Chem. Technol..

[105] Auricchio F., Ferrara A., Morganti S. (2012). Comparison and critical analysis of invariant-based models in relation to their ability to fit human aortic valve data. Ann. Solid Struct. Mech..

[106] Stradins P., Lacis R., Ozolanta I., Purina B., Ose V., Feldmane L., Kasyanov V. (2004). Comparison of biomechanical and structural properties between the human aortic valve and the pulmonary valve. Eur. J. Cardio-Thorac. Surg..

[107] Martin C., Pham T., Sun W. Differences in mechanical properties between human and porcine aortic root. Proceedings of the IEEE Annual International Conference.

[108] Martin C., Pham T., Sun W. (2011). Significant differences in material properties between aged human and porcine aortic tissues. Eur. J. Cardio-Thorac. Surg..

[109] Roach M.R., Burton A.C. (1957). The Reason for the Shape of the Distensibility Curves of Arteries. Can. J. Biochem. Physiol..

[110] Claes E., Atienza J.M., Guinea G.V., Rojo F.J., Bernal J.M., Revuelta J.M., Elices M. Mechanical properties of human coronary arteries. Proceedings of the 2010 Annual International Conference of the IEEE Engineering in Medicine and Biology.

[111] Ozolanta I., Tetere G., Purinya B., Kasyanov V. (1998). Changes in the mechanical properties, biochemical contents and wall structure of the human coronary arteries with age and sex. Med. Eng. Phys..

[112] Holzapfel G.A., Sommer G., Gasser C.T., Regitnig P. (2005). Determination of layer-specific mechanical properties of human coronary arteries with nonatherosclerotic intimal thickening and related constitutive modeling. Am. J. Physiol.-Heart Circ. Physiol..

[113] Vorp D.A., Schiro B.J., Ehrlich M.P., Juvonen T.S., Ergin M.A., Griffith B.P. (2003). Effect of aneurysm on the tensile strength and biomechanical behavior of the ascending thoracic aorta. Ann. Thorac. Surg..

[114] Iliopoulos D.C., Kritharis E.P., Giagini A.T., Papadodima S.A., Sokolis D.P. (2009). Ascending thoracic aortic aneurysms are associated with compositional remodeling and vessel stiffening but not weakening in age-matched subjects. J. Thorac. Cardiovasc. Surg..

[115] Okamoto R.J., Wagenseil J.E., DeLong W.R., Peterson S.J., Kouchoukos N.T., Sundt I.T.M. (2002). Mechanical properties of dilated human ascending aorta. Ann. Biomed. Eng..

[116] Geest J.P.V., Sacks M.S., Vorp D.A. (2004). Age dependency of the biaxial biornechanical Behavior of humian abdominal aorta. J. Biomech. Eng.-Trans. ASME.

[117] Joner M., Finn A.V., Farb A., Mont E.K., Kolodgie F.D., Ladich E., Kutys R., Skorija K., Gold H.K., Virmani R. (2006). Pathology of drug-eluting stents in humans: Delayed healing and late thrombotic risk. J. Am. Coll. Cardiol..

[118] Yang T.H., Kim D.I., Park S.G., Seo J.S., Cho H.J., Seol S.H., Kim S.M., Kim D.K., Kim D.S. (2009). Clinical characteristics of stent fracture after sirolimus-eluting stent implantation. Int. J. Cardiol..

[119] Flege C., Vogt F., Höges S., Jauer L., Borinski M., Schulte V.A., Hoffmann R., Poprawe R., Meiners W., Jobmann M. (2013). Development and characterization of a coronary polylactic acid stent prototype generated by selective laser melting. J. Mater. Sci. Mater. Med..

[120] Ormiston J.A., Serruys P.W. (2009). Bioabsorbable coronary stents. Circ. Cardiovasc. Interv..

[121] Onuma Y., Serruys P.W. (2011). Bioresorbable scaffold: The advent of a new era in percutaneous coronary and peripheral revascularization?. Circulation.

[122] Waksman R. (2006). Biodegradable stents: They do their job and disappear. J. Invasive Cardiol..

[123] Serruys P.W., Chevalier B., Dudek D., Cequier A., DCarrié D., Iniguez A., Dominici M., van der Schaaf R.J., Haude M., Wasungu L. (2015). A bioresorbable everolimus-eluting scaffold versus a metallic everolimus-eluting stent for ischaemic heart disease caused by de-novo native coronary artery lesions (ABSORB II): An interim 1-year analysis of clinical and procedural secondary outcomes from a randomised controlled trial. Lancet.

[124] Gao R., Abizaid A., Banning A., Bartorelli A.L., DŽavík V., Ellis S., Jeong M.H., Legrand V., Spaulding C., Urban P. (2013). One-year outcome of small-vessel disease treated with sirolimus-eluting stents: A subgroup analysis of the e-SELECT registry. J. Interv. Cardiol..

[125] Agrawal C.M., Haas K.F., Leopold D.A., Clark H.G. (1992). Evaluation of poly (L-lactic acid) as a material for intravascular polymeric stents. Biomaterials.

[126] Nuutinen J.P., Clerc C., Reinikainen R., Törmälä P. (2003). Mechanical properties and in vitro degradation of bioabsorbable self-expanding braided stents. J. Biomater. Sci. Polym. Ed..

[127] Bünger C.M., Grabow N., Sternberg K., Kröger C., Ketner L., Schmitz K.P., Kreutzer H.J., Ince H., Nienaber C.A., Klar E. (2007). Sirolimus-eluting biodegradable poly-L-lactide stent for peripheral vascular application: A preliminary study in porcine carotid arteries. J. Surg. Res..

[128] Verheye S., Ormiston J.A., Stewart J., Webster M., Sanidas E., Costa R., Costa J.R., Chamie D., Abizaid A.S., Pinto I. (2014). A next-generation bioresorbable coronary scaffold system: From bench to first clinical evaluation: 6-and 12-month clinical and multimodality imaging results. JACC Cardiovasc. Interv..

[129] Chua S.N.D., MacDonald B.J., Hashmi M.S.J. (2003). Finite element simulation of stent and balloon interaction. J. Mater. Process. Technol..

[130] Lally C., Dolan F., Prendergast P.J. (2005). Cardiovascular stent design and vessel stresses: A finite element analysis. J. Biomech..

[131] Gijsen F.J., Migliavacca F., Schievano S., Socci L., Petrini L., Thury A., Wentzel J.J., van der Steen A.F., Serruys P.W., Dubini G. (2008). Simulation of stent deployment in a realistic human coronary artery. Biomed. Eng. Online.

[132] Imani S.M., Goudarzi A.M., Ghasemi S.E., Kalani A., Mahdinejad J. (2014). Analysis of the stent expansion in a stenosed artery using finite element method: Application to stent versus stent study. Proc. Inst. Mech. Eng. Part H. J. Eng. Med..

[133] Schiavone A., Zhao L.G., Abdel-Wahab A.A. (2014). Effects of material, coating, design and plaque composition on stent deployment inside a stenotic artery—Finite element simulation. Mater. Sci. Eng. C Mater. Biol. Appl..

[134] Clune R., Kelliher D., Robinson J.C., Campbell J.S. (2014). NURBS modeling and structural shape optimization of cardiovascular stents. Struct. Multidiscipl Optim..

[135] Holzapfel G.A., Gasser T.C., Ogden R.W. (2000). A new constitutive framework for arterial wall mechanics and a comparative study of material models. J. Elast. Phys. Sci. Solids.

[136] Holzapfel G.A., Ogden R.W. (2010). Modelling the layer-specific three-dimensional residual stresses in arteries, with an application to the human aorta. J. R. Soc. Interface.

[137] Schiavone A., Qiu T.Y., Zhao L.G. (2017). Crimping and deployment of metallic and polymeric stents—Finite element modelling. Vessel Plus.

[138] Zahedmanesh H., Lally C. (2009). Determination of the influence of stent strut thickness using the finite element method: Implications for vascular injury and in-stent restenosis. Med. Biol. Eng. Comput..

[139] Qiu T.Y., Song M., Zhao L.G. (2018). A computational study of crimping and expansion of bioresorbable polymeric stents. Mech. Time-Depend. Mater..

[140] Nolan D.R., Gower A.L., Destrade M., Ogden R.W., McGarry J.P. (2014). A robust anisotropic hyperelastic formulation for the modelling of soft tissue. J. Mech. Behav. Biomed. Mater..

[141] Migliavacca F., Petrini L., Colombo M., Auricchio F., Pietrabissa R. (2002). Mechanical behavior of coronary stents investigated through the finite element method. J. Biomech..

[142] Karimi A., Razaghi R., Shojaei A., Navidbakhsh M. (2015). An experimental-nonlinear finite element study of a balloon expandable stent inside a realistic stenotic human coronary artery to investigate plaque and arterial wall injury. Biomed. Eng..

[143] Schiavone A., Abunassar C., Hossainy S., Zhao L.G. (2016). Computational analysis of mechanical stress-strain interaction of a bioresorbable scaffold with blood vessel. J. Biomech..

[144] Varcoe R.L., Schouten O., Thomas S.D., Lennox A.F. (2016). Experience with the absorb everolimus-eluting bioresorbable vascular scaffold in arteries below the knee: 12-month clinical and imaging outcomes. JACC Cardiovasc. Interv..

[145] Wang Q., Fang G., Zhao Y., Wang G., Cai T. (2017). Computational and experimental investigation into mechanical performances of Poly-L-Lactide Acid (PLLA) coronary stents. J. Mech. Behav. Biomed. Mater..

[146] Welch T.R., Eberhart R.C., Chuong C.J. (2009). The influence of thermal treatment on the mechanical characteristics of a PLLA coiled stent. J. Biomed. Mater. Res. Part B Appl. Biomater..

[147] Wiebe J., Nef H.M., Hamm C.W. (2014). Current status of bioresorbable scaffolds in the treatment of coronary artery disease. J. Am. Coll. Cardiol..

[148] Cheng G.C., Loree H.M., Kamm R.D., Fishbein M.C., Lee R.T. (1993). Distribution of circumferential stress in ruptured and stable atherosclerotic lesions. A structural analysis with histopathological correlation. Circulation.

[149] Li Z.Y., Howarth S., Trivedi R.A., U-King-Im J.M., Graves M.J., Brown A., Wang L., Gillard J.H. (2006). Stress analysis of carotid plaque rupture based on in vivo high resolution MRI. J. Biomech..

[150] Zhu P., Zhou X., Zhang C., Li H., Zhang Z., Song Z. (2018). Safety and efficacy of ultrathin strut biodegradable polymer sirolimus-eluting stent versus durable polymer drug-eluting stents: A meta-analysis of randomized trials. BMC Cardiovasc. Disord..

[151] Madhavan M.V., Howard J.P., Naqvi A., Ben-Yehuda O., Redfors B., Prasad M., Shahim B., Leon M.B., Bangalore S., Stone G.W. (2021). Long-Term Follow-Up After Ultrathin vs. Conventional 2nd-Generation Drug-Eluting Stents: A Systematic Review and Meta-Analysis of Randomized Controlled Trials. Eur. Heart J..

[152] Palmerini T., Biondi-Zoccai G., Della Riva D., Mariani A., Sabaté M., Smits P.C., Kaiser C., D’Ascenzo F., Frati G., Mancone M. (2014). Clinical Outcomes with Bioabsorbable Polymer- versus Durable Polymer-Based Drug-Eluting and Bare-Metal Stents:Evidence from a Comprehensive Network Meta-Analysis. J. Am. Coll. Cardiol..

[153] Bangalore S., Toklu B., Amoroso N., Fusaro M., Kumar S., Hannan E.L., Faxon D.P., Feit F. (2013). Bare Metal Stents, Durable Polymer Drug Eluting Stents, and Biodegradable Polymer Drug Eluting Stents for Coronary Artery Disease: Mixed Treatment Comparison Meta-Analysis. BMJ.

[154] Kolandaivelu K., Swaminathan R., Gibson W.J., Kolachalama V.B., Nguyen-Ehrenreich K.-L., Giddings V.L., Coleman L., Wong G.K., Edelman E.R. (2011). Stent Thrombogenicity Early in High-Risk Interventional Settings Is Driven by Stent Design and Deployment and Protected by Polymer-Drug Coatings. Circulation.

[155] Pilgrim T., Piccolo R., Heg D., Roffi M., Tuller D., Muller O., Moarof I., Siontis G.C.M., Cook S., Weilenmann D. (2018). Ultrathin-Strut, Biodegradable-Polymer, Sirolimus-Eluting Stents versus Thin-Strut, Durable-Polymer, Everolimus-Eluting Stents for Percutaneous Coronary Revascularisation: 5-Year Outcomes of the BIOSCIENCE Randomised Trial. Lancet.

[156] Piccolo R., Heg D., Franzone A., Roffi M., Tuller D., Vuilliomenet A., Muller O., Cook S., Weilenmann D., Kaiser C. (2016). Biodegradable-Polymer Sirolimus-Eluting Stents Versus Durable-Polymer Everolimus-Eluting Stents in Patients With Acute ST-Segment Elevation Myocardial Infarction: Insights From the 2-Year Follow-Up of the BIOSCIENCE Trial. JACC Cardiovasc. Interv..

[157] Pilgrim T., Piccolo R., Heg D., Roffi M., Tuller D., Vuilliomenet A., Muller O., Cook S., Weilenmann D., Kaiser C. (2016). Biodegradable Polymer Sirolimus-Eluting Stents versus Durable Polymer Everolimus-Eluting Stents for Primary Percutaneous Coronary Revascularisation of Acute Myocardial Infarction. EuroIntervention.

[158] Lemos P.A., Chandwani P., Saxena S., Ramachandran P.K., Abhyankar A., Campos C.M., Marchini J.F., Galon M.Z., Verma P., Sandhu M.S. (2016). Clinical Outcomes in 995 Unselected Real-World Patients Treated with an Ultrathin Biodegradable Polymer-Coated Sirolimus-Eluting Stent: 12-Month Results from the FLEX Registry. BMJ Open.

[159] Zaman A., deWinter R.J., Kogame N., Chang C.C., Modolo R., Spitzer E., Tonino P., Hofma S., Zurakowski A., Smits P.C. (2019). Safety and Efficacy of a Sirolimus-Eluting Coronary Stent with Ultra-Thin Strut for Treatment of Atherosclerotic Lesions (TALENT): A Prospective Multicentre Randomised Controlled Trial. Lancet.

[160] Lowe H.C., Oesterle S.N., Khachigian L.M. (2002). Coronary in-stent restenosis: Current status and future strategies. J. Am. Coll. Cardiol..

[161] Timmins L.H., Miller M.W., Clubb F.J., Moore J.E. (2011). Increased artery wall stress post-stenting leads to greater intimal thickening. Lab. Investig..

[162] Haga J.H., Li Y.S., Chien S. (2007). Molecular basis of the effects of mechanical stretch on vascular smooth muscle cells. J. Biomech..

[163] Chung I.M., Gold H.K., Schwartz S.M., Ikari Y., Reidy M.A., Wight T.N. (2002). Enhanced extracellular matrix accumulation in restenosis of coronary arteries after stent deployment. J. Am. Coll. Cardiol..

[164] Sharkawi T., Cornhill F., Lafont A., Sabaria P., Vert M. (2007). Intravascular bioresorbable polymeric stents: A potential alternative to current drug eluting metal stents. J. Pharm. Sci..

[165] Pauck R.G., Reddy B.D. (2015). Computational analysis of the radial mechanical performance of PLLA coronary artery stents. Med. Eng. Phys..

[166] Möller D., Reimers W., Pyzalla A., Fischer A. (2001). Residual stresses in coronary artery stents. J. Biomed. Mater. Res..

[167] Debusschere N., Segers P., Dubruel P., Verhegghe B., De Beule M. (2015). A finite element strategy to investigate the free expansion behaviour of a biodegradable polymeric stent. J. Biomech..

[168] Eshghi N., Hojjati M.H., Imani M., Goudarzi A.M. (2011). Finite element analysis of mechanical behaviors of coronary stent. Proc. Eng..

[169] Garcia-Garcia H.M., Serruys P.W., Campos C.M., Muramatsu T., Nakatani S., Zhang Y.J., Onuma Y., Stone G.W. (2014). Assessing bioresorbable coronary devices: Methods and parameters. JACC Cardiovasc. Interv..

[170] Yamaji K., Brugaletta S., Sabaté M., Iñiguez A., Jensen L.O., Cequier A., Hofma S.H., Christiansen E.H., Suttorp M., van Es G.A. (2017). Effect of Post-Dilatation Following Primary PCI With Everolimus-Eluting Bioresorbable Scaffold Versus Everolimus-Eluting Metallic Stent Implantation: An Angiographic and Optical Coherence Tomography TROFI II Substudy. JACC Cardiovasc. Interv..

[171] Ali Z.A., Karimi Galougahi K., Shlofmitz R., Maehara A., Mintz G.S., Abizaid A., Chamié D., Hill J., Serruys P.W., Onuma Y. (2018). Imaging-guided pre-dilatation, stenting, post-dilatation: A protocolized approach highlighting the importance of intravascular imaging for implantation of bioresorbable scaffolds. Expert Rev. Cardiovasc. Ther..

[172] Kumar A., Bhatnagar N. (2021). Finite element simulation and testing of cobalt-chromium stent: A parametric study on radial strength, recoil, foreshortening, and dogboning. Comput. Methods Biomech. Biomed. Eng..

[173] Kratochvíl J. (1973). On a finite strain theory of elastic-inelastic materials. Acta Mech..

[174] Bodke M., Shubham A.A.J., Anup S. (2026). Influence of matrix inelasticity on the mechanical properties of bioinspired composites. Bioinspir. Biomim..

[175] Kuetzing P.M., Scheven U.M., Arruda E.M. (2026). Submaximal low-strain cyclic loading induces localized inelastic deformation & diminished energy dissipation in the anterior cruciate ligament. J. Mech. Behav. Biomed. Mater..

[176] Gharleghi R., Wright H., Luvio V., Jepson N., Luo Z., Senthurnathan A., Babaei B., Prusty B.G., Ray T., Beier S. (2021). A multi-objective optimization of stent geometries. J. Biomech..

[177] Karanasiou G.S., Papafaklis M.I., Conway C., Michalis L.K., Tzafriri R., Edelman E.R., Fotiadis D.I. (2017). Stents: Biomechanics, Biomaterials, and Insights from Computational Modeling. Ann. Biomed. Eng..

[178] Soares S.G., Moore E.J. (2016). Biomechanical Challenges to Polymeric Biodegradable Stents. Annals of Biomedical Engineering. Med. Stents State Art. Future Dir..

[179] Muliana A., Rajagopal K.R. (2012). Modeling the response of nonlinear viscoelastic biodegradable polymeric stents. Int. J. Solids Struct..

[180] Syaifudin A., Takeda R., Sasaki K. (2018). Development of asymmetric stent for treatment of eccentric plaque. Biomed. Mater. Eng..

[181] Ribeiro N.S., Folgado J., Rodrigues H.C. (2021). Surrogate-based multi-objective design optimization of a coronary stent: Altering geometry toward improved biomechanical performance. Int. J. Numer. Methods Biomed. Eng..

[182] Blair R.W., Dunne N.J., Lennon A.B., Menary G.H. (2019). Multi-objective optimisation of material properties and strut geometry for poly(L-lactic acid) coronary stents using response surface methodology. PLoS ONE.

[183] Balossino R., Gervaso F., Migliavacca F., Dubini G. (2008). Effects of different stent designs on local hemodynamics in stented arteries. J. Biomech..

[184] Pant S., Limbert G., Curzen N.P., Bressloff N.W. (2011). Multiobjective design optimisation of coronary stents. Biomaterials.

[185] Li Y., Wang J., Sheng K., Miao F., Wang Y., Zhang Y., Hou R., Mei D., Sun Y., Zheng Y. (2022). Optimizing structural design on biodegradable magnesium alloy vascular stent for reducing strut thickness and raising radial strength. Mater. Des..

[186] Wu W., Petrini L., Gastaldi D., Villa T., Vedani M., Lesma E., Previtali B., Migliavacca F. (2010). Finite element shape optimization for biodegradable magnesium alloy stents. Ann. Biomed. Eng..

[187] Chen C., Chen J., Wu W., Shi Y., Jin L., Petrini L., Shen L., Yuan G., Ding W., Ge J. (2019). In vivo and in vitro evaluation of a biodegradable magnesium vascular stent designed by shape optimization strategy. Biomaterials.

[188] Grogan J.A., Leen S.B., McHugh P.E. (2013). Optimizing the design of a bioabsorbable metal stent using computer simulation methods. Biomaterials.

[189] Bozsak F., Gonzalez-Rodriguez D., Sternberger Z., Belitz P., Bewley T., Chomaz J.-M., I Barakat A. (2015). Optimization of Drug Delivery by Drug-Eluting Stents. PLoS ONE.

[190] Li H., Liu K., Zhao D., Wang M., Li Q., Hou J. (2018). Multi-Objective Optimizations for Microinjection Molding Process Parameters of Biodegradable Polymer Stent. Materials.

[191] Blouza A., Dumas L., M’Baye I. Multiobjective optimization of a stent in a fluid-structure context. Proceedings of the 10th Annual Conference Companion on Genetic and Evolutionary Computation.

[192] Nappi F., Spadaccio C., Al-Attar N., Acar C. (2015). The Ross procedure at the crossroads: Lessons from biology: Is Dr Ross’s dream concluded?. Int. J. Cardiol..

[193] Nappi F., Spadaccio C., Chello M., Acar C. (2015). The Ross procedure: Underuse or under-comprehension?. J. Thorac. Cardiovasc. Surg..

[194] Nappi F., Spadaccio C., Fraldi M. (2016). Reply: Papillary Muscle Approximation Is an Anatomically Correct Repair for Ischemic Mitral Regurgitation. J. Am. Coll. Cardiol..

[195] Rama A., Nappi F., Praschker B.G., Gandjbakhch I. (2008). Papillary muscle approximation for ischemic mitral valve regurgitation. J. Card. Surg..

